# Synthesis and Photobehavior of a New Dehydrobenzoannulene-Based HOF with Fluorine Atoms: From Solution to Single Crystals Observation

**DOI:** 10.3390/ijms22094803

**Published:** 2021-04-30

**Authors:** Eduardo Gomez, Ichiro Hisaki, Abderrazzak Douhal

**Affiliations:** 1 Physical Chemistry Department, Faculty of Environmental Sciences and Biochemistry, and INAMOL, University of Castilla-La Mancha, Avenida Carlos III, S.N., 45071 Toledo, Spain; eduardo.gomez@uclm.es; 2 Division of Chemistry, Graduate School of Engineering Science, Osaka University, 1-3 Machikaneyama, Toyonaka, Osaka 560-8531, Japan

**Keywords:** hydrogen-bonded organic frameworks (HOFs), spectroscopy, fluorescence microscopy

## Abstract

Hydrogen-bonded organic frameworks (HOFs) are the focus of intense scientific research due their potential applications in science and technology. Here, we report on the synthesis, characterization, and photobehavior of a new HOF (T12F-1(124TCB)) based on a dehydrobenzoannulene derivative containing fluorine atoms (T12F-COOH). This HOF exhibits a 2D porous sheet, which is hexagonally networked via H-bonds between the carboxylic groups, and has an interlayers distance (4.3 Å) that is longer than that of a typical π–π interaction. The presence of the fluorine atoms in the DBA molecular units largely increases the emission quantum yield in DMF (0.33, T12F-COOH) when compared to the parent compound (0.02, T12-COOH). The time-resolved dynamics of T12F-COOH in DMF is governed by the emission from a locally excited state (S1, ~0.4 ns), a charge-transfer state (S1(CT), ~2 ns), and a room temperature emissive triplet state (T1, ~20 ns), in addition to a non-emissive triplet structure with a charge-transfer character (T1(CT), τ = 0.75 µs). We also report on the results using T12F-ester. Interestingly, FLIM experiments on single crystals unravel that the emission lifetimes of the crystalline HOF are almost twice those of the amorphous ones or the solid T12F-ester sample. This shows the relevance of the H-bonds in the photodynamics of the HOF and provides a strong basis for further development and study of HOFs based on DBAs for potential applications in photonics.

## 1. Introduction

Hydrogen-bonded organic frameworks (HOFs) have emerged as an exciting class of materials constructed entirely from organic molecules connected mainly by hydrogen-bonds (H-bonds), in addition to π–π interactions [1,2,3]. Depending on the organic linker, these materials can have different pore sizes and crystalline structures, making them very attractive for several applications, such as gas storage and separation, proton conduction, explosive detection, and optoelectronic devices, among others [4,5,6,7,8,9,10]. Nowadays, the possibility of creating new HOFs with larger pore sizes to encapsulate big molecules (i.e., drugs) is a research area of great interest [11,12]. For example, the HOF named PFC-1 has been shown to efficiently encapsulate doxorubicin for synergistic chemo-photodynamic therapy [13]. On other hand, several HOF materials exhibit interesting fluorescence changes when interacting with smaller (HCl, iodine molecule, or metal ions) or bigger (benzene, toluene, aniline, etc.) aromatic molecules [7,9,10,14,15,16,17].

HOF materials based on dehydrobenzoannulene (DBA) derivatives are under intense study because of their interesting chemical and spectroscopic properties, which can be used in lighting, sensing or imaging [18,19,20,21,22]. For example, the T12-apo HOF displays a long fluorescence lifetime (25 ns) and optimal CIE coordinates (0.42, 0.55), which make it a candidate for the fabrication of WLEDs [8]. The Ex-apo HOF, which can be considered as the second generation of T12-apo, has a larger pore size to encapsulate big molecules, and it exhibits a rich fluorescence behaviour [22,23,24]. Therefore, we combined the pore size of HOFs based on DBAs with fluorine-substituted ligands with the aim of obtaining a new fluorescent HOF with a different pore size.

**Figure 1:**
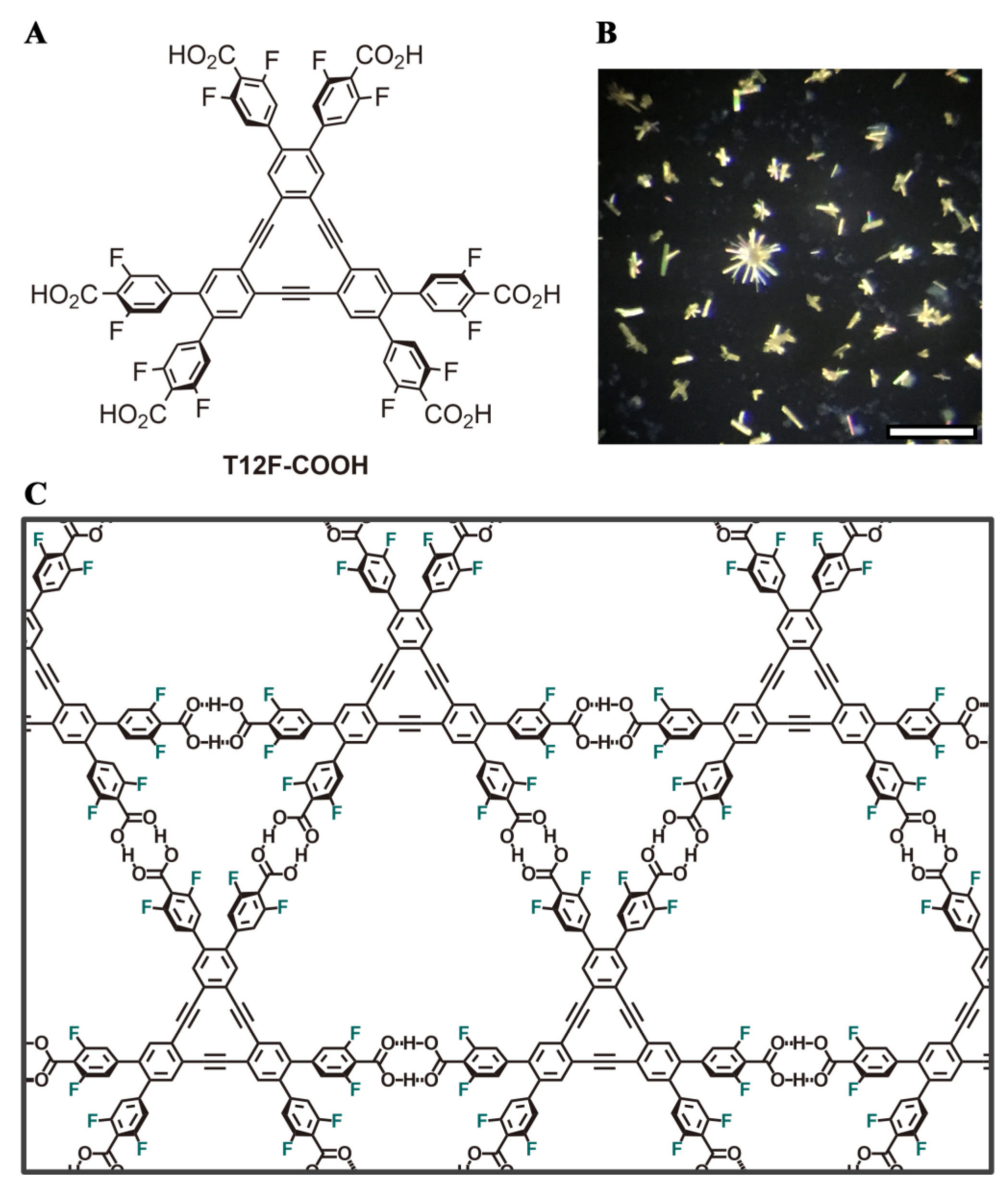
(**A**) Molecular structure of T12F-COOH. (**B**) Polarized optical microscope images of the hydrogen-bonded organic framwork (HOF) crystals. Scale bar: 100 μm (**C**) Illustration of the H-bond interactions between the molecular units producing the T12F-1(124TCB) HOF.

Herein, we report on the synthesis and spectroscopic properties of two new DBA derivatives (T12F-COOMe and T12F-COOH) in N-N-dimethylformamide (DMF) solution (Figure 1). The crystallization of the latter makes the production of a new HOF possible; this new HOF, T12F-1(124TCB), exhibits a 2D hexagonally networked, porous sheet via H-bonds between the carboxylic groups, and an interlayers distance (4.3 Å) longer than that of a typical π–π interaction (3.5 Å). Starting with the ligands in DMF, the steady-state experiments reveal that the first allowed transition is the S_0_→S_2_, due to the S_0_→S_1_ transition is forbidden.

Upon electronic excitation, both molecular units undergo an ultrafast intramolecular charge transfer (ICT) process in less than 15 ps to yield a charge-transfer structure fluorescing in the ns regime and phosphorescing in short (ns) and long (µs) regimes. The phosphorescence signals reflect the emission from the triplet states corresponding to the singlet ones of the initially populated and ICT states, respectively. For the HOF sample, the experiments using fluorescence lifetime imaging (FLIM) under the microscope show two kinds of structures: one of a well-crystallized HOF (T12F-1(124TCB)), which displays two fluorescence lifetimes of 5.3 and 21.3 ns, and a cotton-like aspect, not fully crystallized with a soft and amorphous structure, decaying with fluorescence lifetimes of 2.7 and 12.6 ns. These values are comparable to those exhibited by the ester (amorphous) sample, T12F-ester (3.2 and 14 ns). The emission quantum yield of T12F-COOH (0.33) is remarkably higher than that of T12-COOH (0.02), in agreement with our expectations.

## 2. Results

### 2.1. Synthesis of the Compounds

The Suzuki–Miyaura cross-coupling of ((2-azido-4,5-dibromophenyl)ethynyl)triisopropylsilane (**1**) and 3,5-difluoro-4-methoxycarbonylphenylboronic acid pinacolyl ester leads to **2**, which was treated with methyl iodide to give **3**. Desilylation followed by cyclotrimerization through tSonogashira reaction of **3** gave T12F-COOMe, which, after hydrolysis, gave T12F-COOH (Scheme S1). The synthesized compound T12F-1(124TCB) was recrystallized by slow evaporation of a *N,N*-dimethylformamide and 1,2,4-trichlorobenzene solution at 80 °C.

### 2.2. Crystal Structure Description of the HOF T12F-1(124TCB)

**Figure 2:**
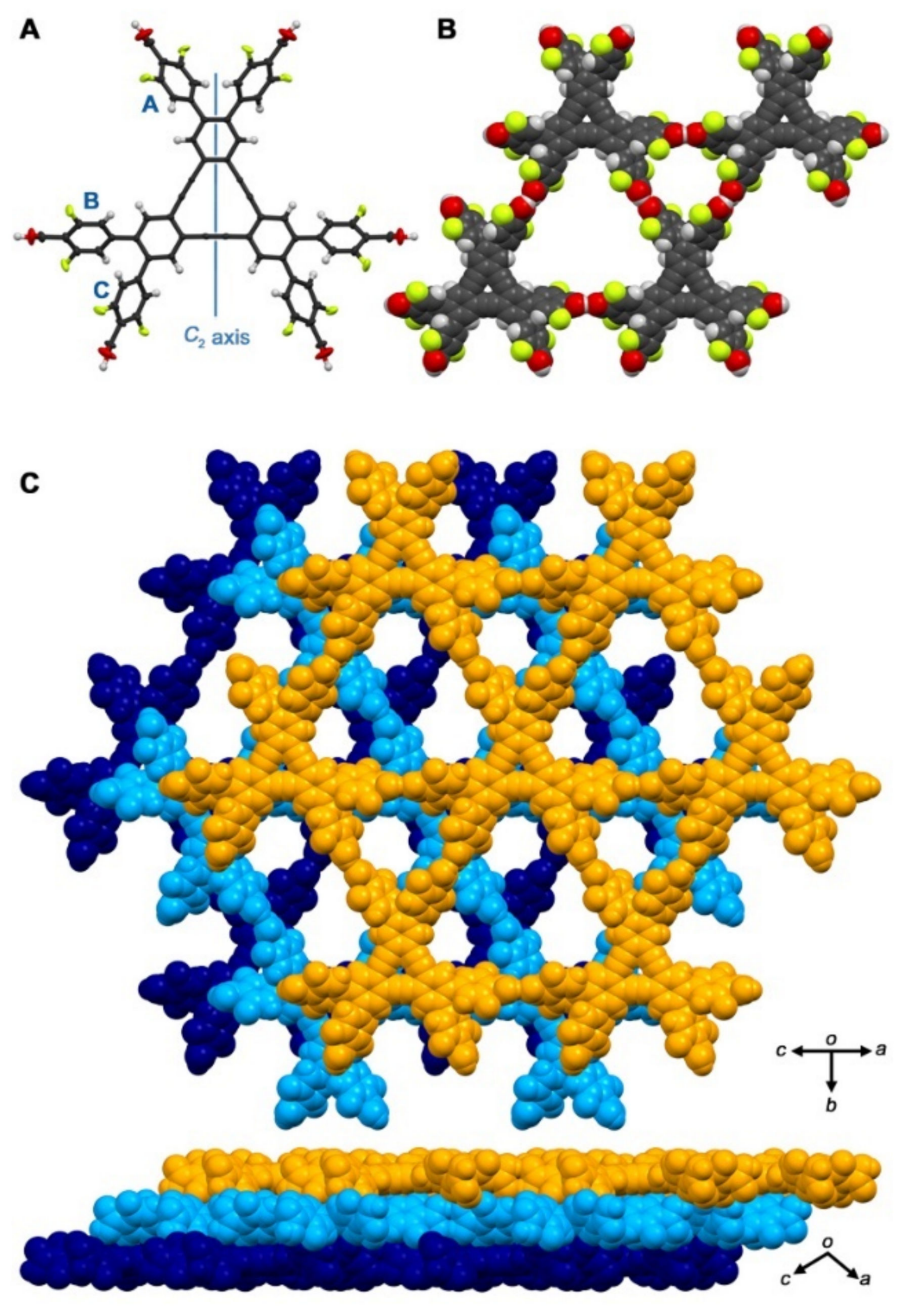
Crystal structure of hydrogen-bonded organic framework (HOF) <T12F-1(124TCB). (**A**) *C*_2_-symmetric molecular structure drawn by anisotropic displacement ellipsoid plot with 50% probability. (**B**) Packing motif of the framework with two kinds of different pores. (**C**) Selected layered structure of the HOF.

T12F-COOH crystallizes in space group *I*2/*a*. The crystal structure of T12F-1(124TCB) is shown in Figure 2.

The molecule has a *C*_2_-symmetric axis in the plane of the DBA ring (Figure 2A and Figure S1). The DBA core is highly planar; the root mean square deviation (RMSD) value of the core is calculated to be 0.047 Å. The molecular structure observed in the crystal indicates that the peripheral phenylene arms, on the other hand, are twisted against the central DBA core; the dihedral angles between the peripheral phenyl rings (A, B, and C) and the annulene core are +51.0°, −46.1°, and −50.6°, respectively (Table 1). The carboxylic groups are also twisted by 32.1° to 40.7° against the phenylene ring due to steric hindrance originating from the fluorine atoms in the *ortho*-positions of the carboxylic groups. Consequently, the carboxylic groups are nearly perpendicular to the plane of the central DBA core.

T12F-COOH forms a two-dimensional, hexagonally-networked porous sheet via typical hydrogen bonded dimerization of the carboxylic groups (Figure 2). The sheet contains two kinds of voids with smaller triangular and larger hexagonal apertures. The sheet is slip-stacked to overlap the rhombic parts of the stacked frameworks (Figure S2A). The annulene rings in the adjacent layers do not interact directly with each other, except for small stacking between the annulated benzene rings, while they interact with the phenylene rings as shown in Figure S2B. The distance between the adjacent layers is 4.278 Å, which is clearly longer than that of a typical π–π interaction. In the framework, void channels with an aperture size of 17 × 8 Å^2^ are formed along the *a* axis, and the ratio of accessible void volume to solvent molecules is calculated to be 53% (Figure S3). Thus, solvent molecules are accommodated in these channels. One of them was crystallographically solved to be disordered in two positions in the triangular channels, while the others were not due to severe disorder (Figure S4).

### 2.3. Spectroscopic Properties

#### 2.3.1. Steady-State Absorption and Emission Studies in DMF Solution

**Figure 3:**
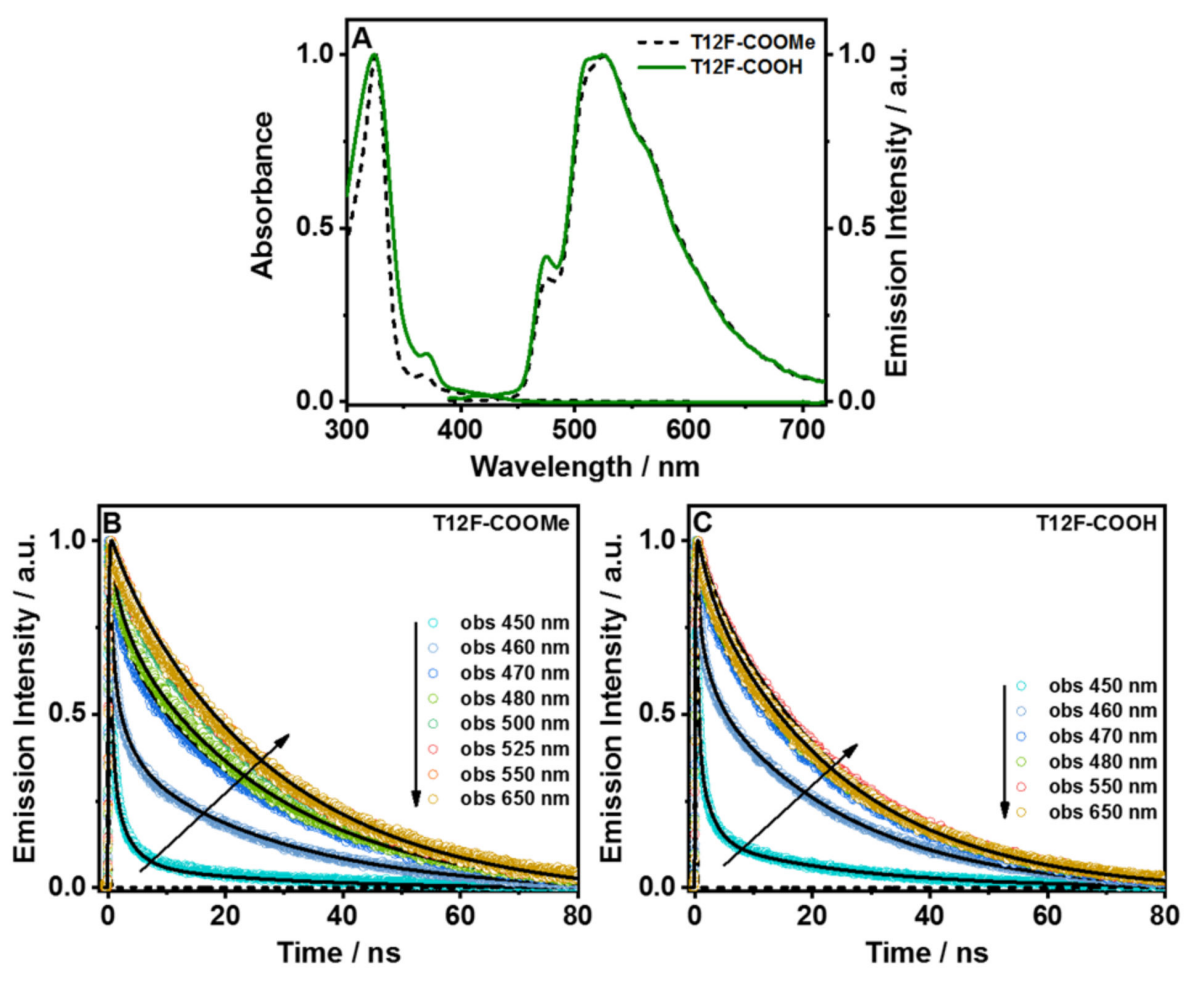
(**A**) Steady-state absorption and emission spectra of T12F-COOMe and T12F-COOH in DMF solution. The excitation wavelength was 370 nm. Magic-angle emission decays of (**B**) T12F-COOMe and (**C**) T12F-COOH in DMF solution upon excitation at 390 nm. The solid lines are from the best global fit using a multiexponential function.

To begin with, we characterized the spectroscopic properties of the linker, T12F-COOH, and its methylated partner (T12F-COOMe) in DMF solution. The UV-visible absorption spectra are dominated by a band with a maximum intensity at 321 nm and a shoulder at 370 nm (Figure 3A). From our previous studies on DBA derivatives with the same main core, T12-COOMe and T12-COOH, we have assigned this absorption band (325 nm) to the S_0_→S_2_ transition (Figure S5) [25]. The difference between these molecules and the ones in this report is the fluorine atoms in the *ortho*-position relative to the carboxylic acids. Thus, we assign the intense 321 nm band to the S_0_→S_2_ transition and the shoulder to a weak S_0_→S_1_ transition.

The emission spectra of both molecules show very similar and single broad bands with an intensity maximum at 525 nm, in addition to two shoulders at 475 and 511 nm (Figure 3A). Similar behavior has been observed in T12-COOMe and T12-COOH [25]. While the methylated ester partners (T12-COOMe and T12F-COOMe) display the same behavior, the protonated ones differ a lot. The emission spectrum of T12F-COOH is more like the T12-COOMe spectrum, which suggests a significantly weaker interaction between T12F-COOH and DMF molecules than in T12-COOH/DMF, where we observed the formation of the anionic structure (Figure S12). This difference reflects the inductive effect of the fluorine atoms in T12F-COOH, which favors the ICT process between the core and the carboxylic acids, making the latter less reactive with DMF molecules. A dependence of the photoinduced charge/proton transfer efficiency on the position or insertion of the substituting group in the aromatic ring has been reported for other aromatic systems [26,27,28].

The excitation spectra of T12F-COOMe and T12F-COOH in DMF solution at different observation wavelengths are similar in shape and position to the absorption spectra, indicating a common ground-state origin for the excited species (Figure S6).

The fluorescence quantum yields (ϕ_f_) of T12F-COOMe and T12F-COOH in DMF solution are 0.40 and 0.33, respectively. These values are quite high, although there are still efficient non-radiative channels, which are the main relaxation pathways. This will be examined in the nanosecond (ns) flash photolysis study. T12-COOMe and T12-COOH in DMF solution show ϕ_f_ values of 0.29 and 0.02, respectively [25]. Clearly, the large change (15 times) in ϕ_f_ value for T12F-COOH is caused by the presence of fluorine atoms in the molecular structure, which affects the interaction with DMF molecules and thus the resulting emitting structure.

#### 2.3.2. Picosecond Time-Resolved Experiments in DMF Solution

To characterize the photodynamical behavior of the excited molecules, we carried out ps-TCSPC experiments. Figure 3B,C show the emission decay of T12F-COOMe and T12F-COOH, respectively, in a DMF solution, upon excitation at 371 nm. Table 2 gives the time constants, pre-exponential factors, and their relative contributions obtained from a global multiexponential fit.

For both molecules, we found three time components that are decaying through the whole observation spectral range, with values of 470 ps, 2.5 ns, and 23.1 ns for T12F-COOMe, and 400 ps, 2.0 ns, and 21.4 ns for T12F-COOH. Thus, both samples exhibit very similar time constants for their emission decays, in agreement with their similar fluorescent quantum yields (0.40 and 0.33, respectively). The shortest component is present only at the bluest side of the emission spectrum (450 and 480 nm), while the other two components contribute to the dynamics across the whole spectral range. The ~2 ns component has its highest contribution at the bluest part of the emission spectrum, while the ~22 ns one is more dominant at the reddest region. The spectral behavior of the short-time component indicates that it arises from the locally excited state of both molecules, while the ~2 ns one reflects the emission of species with a CT character after undergoing an ICT reaction, which is shorter than the resolution of the used setup (~15 ps). Our previous report on T12-COOMe and T12-COOH in DMF solution showed the occurrence of two ICT processes: one within the aromatic core (whose CT species decay in 1.1 ns), and another from the phenyl groups to the core (whose CT species decay in 4.5 ns) [25]. However, from the results of this report (T12F-COOMe and T12F-COOH), we observed only one component of ~2 ns. The 12 fluorine atoms insertion on the phenyl rings carrying the carboxylic groups produces strong electronegative sites, which induce a large charge redistribution and affect the opening of ICT pathways. Several studies have reported on the change in charge- and proton-transfer reaction dynamics upon the insertion of a substituent in aromatic molecules [26,27,28,29,30]. Finally, the longest component (~22 ns) has a value comparable to the ones reported for other DBA derivatives assigned to emission from the triplet state [25]. To further confirm its origin, we carried out ps experiments on T12F-COOMe and T12F-COOH in DMF, purged with molecular oxygen (Figure S7). The obtained decays of the samples in a saturated oxygen atmosphere are clearly shorter than those equilibrated with air (Figure S8). For example, the long component shows a clear decrease (from 21.4 ns to 15.6 ns for T12F-COOH, and from 23.1 ns to 19.1 ns for T12F-COOMe), confirming that this emission comes from the triplet state (T_1_). The other components remain practically unaffected for both molecules, indicating that they reflect lifetimes of singlet states (Table S2).

**Scheme 1:**
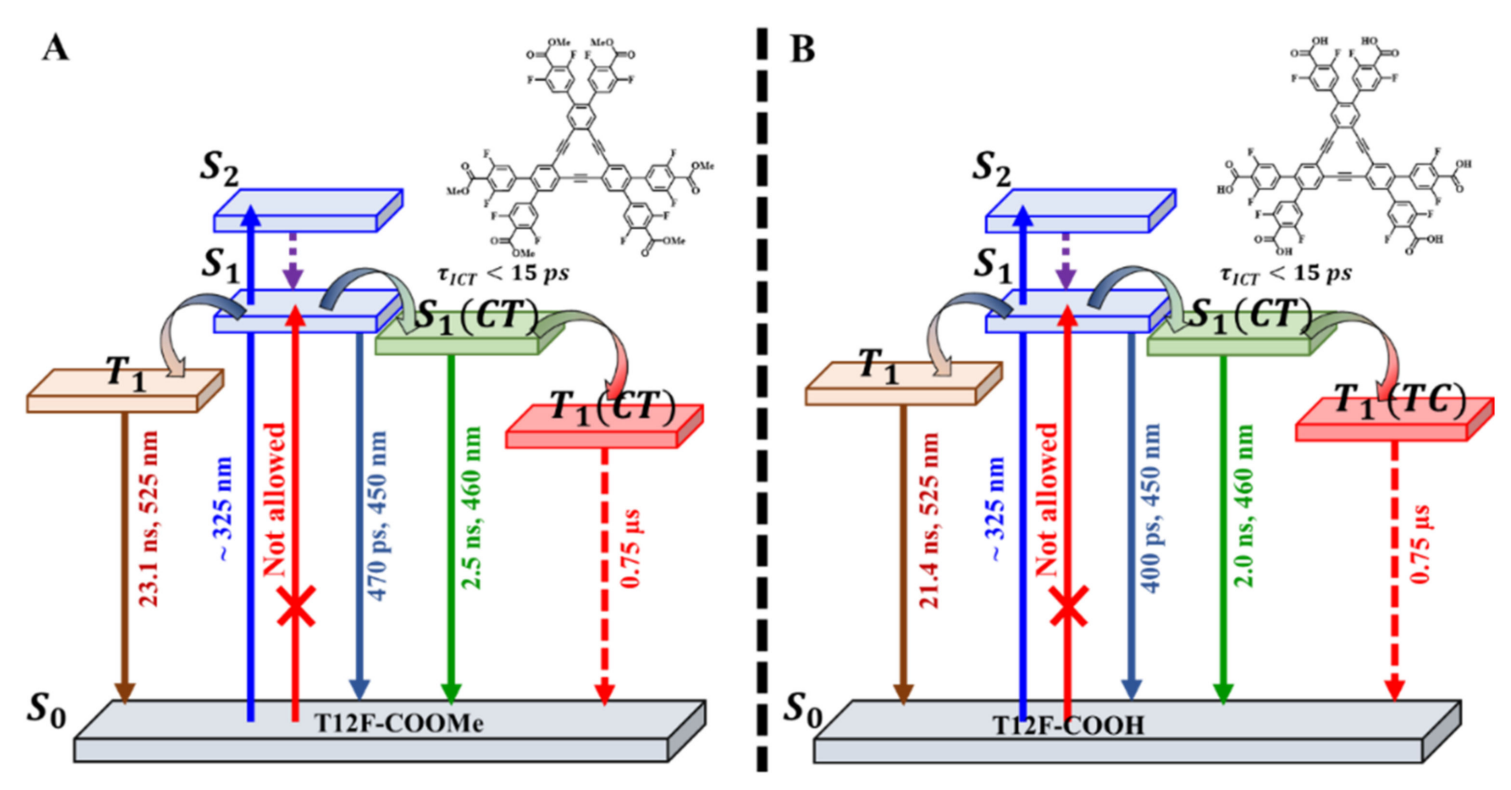
Photophysical diagram (not to scale) showing the time constants, spectral position intensity maxim along of the related electronic transitions, involved events, and emissive states of (**A**) T12F-COOMe and (**B**) T12F-COOH in DMF solution.

Scheme 1 illustrates the observed photobehavior and summarizes the above discussion for both molecules in DMF solution. The wavelength values of the emission spectra were estimated from the contribution of each species to the emission decays, combined with the steady-state emission spectrum.

#### 2.3.3. Nanosecond Flash Photolysis Studies in DMF Solution

**Figure 4:**
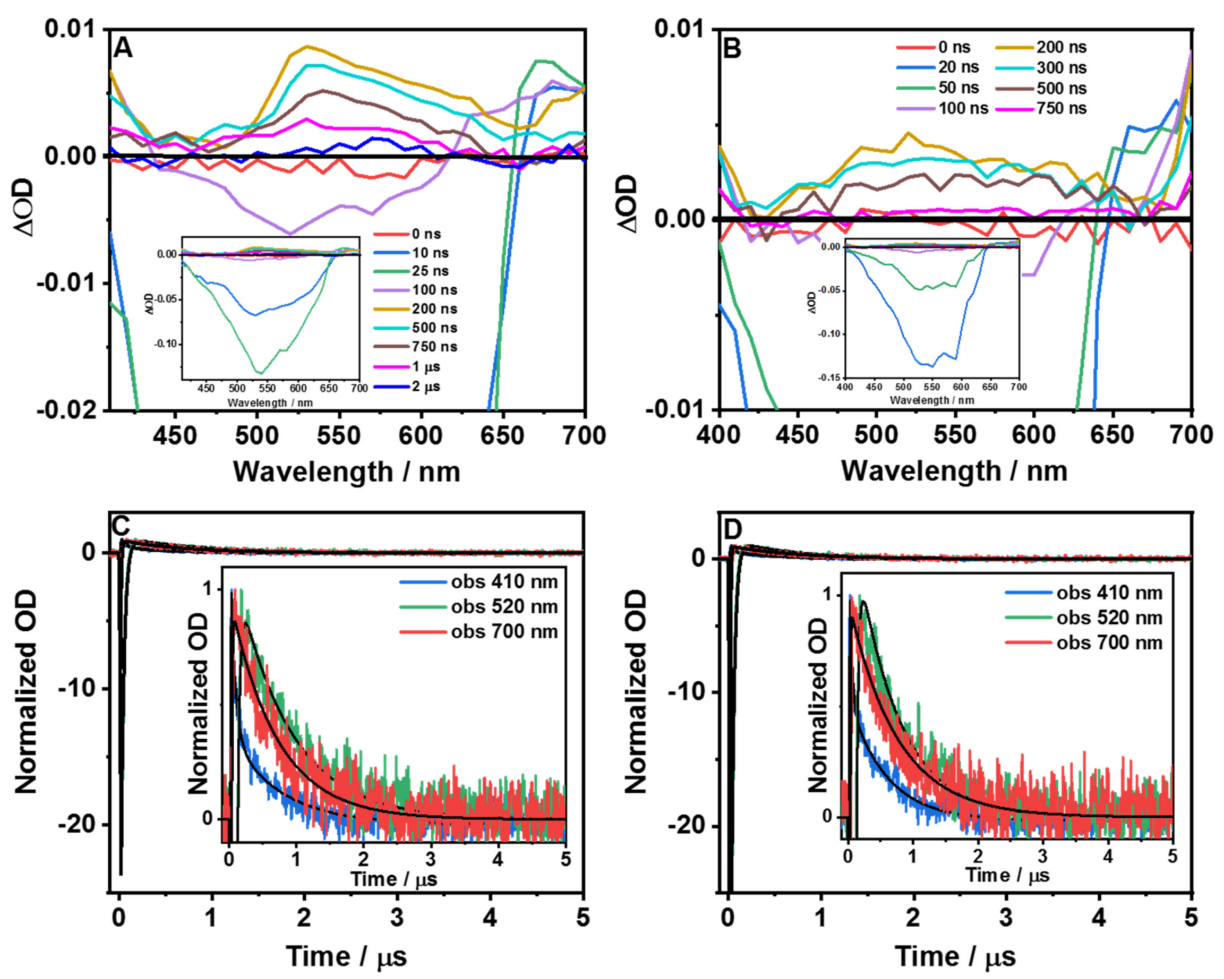
Time-resolved transient absorption spectra at different delay times (ns–µs) upon excitation at 355 nm of (**A**) T12F-COOMe and (**B**) T12F-COOH in DMF solution. Transient decays (µs regime) under atmospheric conditions (air) at the indicated probing wavelengths of (**C**) T12F-COOMe and (**D**) T12F-COOH in DMF solution. The solid lines are from the best fit using a multiexponential function.

To study the slow dynamics of both molecules, we used a ns flash photolysis technique (Figure 4). For T12F-COOMe in DMF, Figure 4A shows representative transient absorption (TA) spectra at different delay times, while Figure 4C displays the related decays under atmospheric conditions. Figure S9 shows the TA decays of the sample saturated with molecular oxygen. Table 3 contains the time constants (τ_i_) and preexponential factors (a_i_ normalized to 100) obtained from multiexponential fits of the related signals. The TA spectra at short delay times (<200 ns) show an intense negative band at ~520 nm and a weak one at ~675 nm, while at longer delay times (>200 ns), the negative band becomes positive and splits into two, and later into three bands, with intensity maxima at ~420, ~530, and ~700 nm. Comparing these results with the one from the steady-state observation, we suggest that the negative band corresponds to the emission from the triplet state. Gating at 520 nm, the decay time constants under air and oxygen atmospheres are τ_1_ = 30 ns and τ_2_ = 0.79 µs, and τ_1_ = 10 ns and τ_2_ = 0.12 µs, respectively. The short component is negative in sign, which indicates a radiative process, and is assigned to the phosphorescence of T_1_, as we observed in the ps TCSPC experiments. This T_1_ corresponds to S_1_ of the locally populated states. On the other hand, the longest component, with a positive signal, reflects the absorption of another triplet state, T_1_(CT), originating from the S_1_(CT) structure formed after an ICT in the excited species. Thus, we suggest that this triplet state has a charge-transfer character as its parent S_1_(CT).

While we observed similar behavior upon gating at 700 nm, probing at 410 nm led to a different result (Figure 4C). Now, the shortest component under air and oxygen atmospheres is positive in sign, and the time constants are τ_1_ = 35 ns, τ_2_ = 0.76 µs, and τ_1_ = 15 ns and τ_2_ = 0.11 µs, respectively. We suggest that the short and long components reflect the decay time of the absorption signals of the T_1_ and T_1_(CT) structures, in agreement with the above observation at other probed wavelengths (Scheme 1).

For T12F-COOH in DMF, Figure 4B shows the TA spectra at different delay times, while Figure 4D displays the decay under atmospheric conditions at different observation wavelengths. Figure S9 shows these results for a sample under oxygen atmosphere. The spectra at short delay times (<100 ns) exhibit negative (~550 nm) and positive (~675 nm) bands, while at longer times, we observed positive bands: ~400 nm, ~520 nm, and ~675 nm. Based on the assignment of the behavior of T12F-COOMe, we attribute the negative band to the phosphorescence of T_1_. Looking at the time constants when gating at 520 nm under air (τ_1_ = 28 ns and τ_2_ = 0.72 µs) and oxygen (τ_1_ = 20 ns and τ_2_ = 0.15 µs) conditions, we suggest the same scenario for the methylated ester sample; τ_1_ and τ_2_ are the lifetimes of T_1_ and T_1_(CT) of T12F-COOH in DMF. The decay at 410 nm gives 35 ns and 16 ns time constants, under air and oxygen atmosphere conditions, respectively, supporting the above assignment of the ns-T_1_ state, which emits at room temperature.

Scheme 1 displays the electronic energy diagrams of T12F-COOMe and T12F-COOH in DMF, involving the S_0_, S_1_, S_1_(CT), T_1_ and T_1_(CT) states, the related time constants of their decays, and the estimated wavelengths of their absorption and emission spectra.

#### 2.3.4. Time-Resolved Confocal Fluorescence Microscopy Experiments

After deciphering the spectroscopic properties of both molecules in solution, we performed time-resolved confocal fluorescence microscopy measurements upon excitation at 390 nm of the materials in a solid state formed by both molecules. It is important to note that while the material T12F-ester (formed by T12F-COOMe) displays an amorphous structure and the force that binds the molecules in it are mainly π–π interactions, the T12F-1(124TCB) HOF is formed thanks to H-bonding and π–π interactions between the T12F-COOH molecular units (Figure 1).

**Figure 5:**
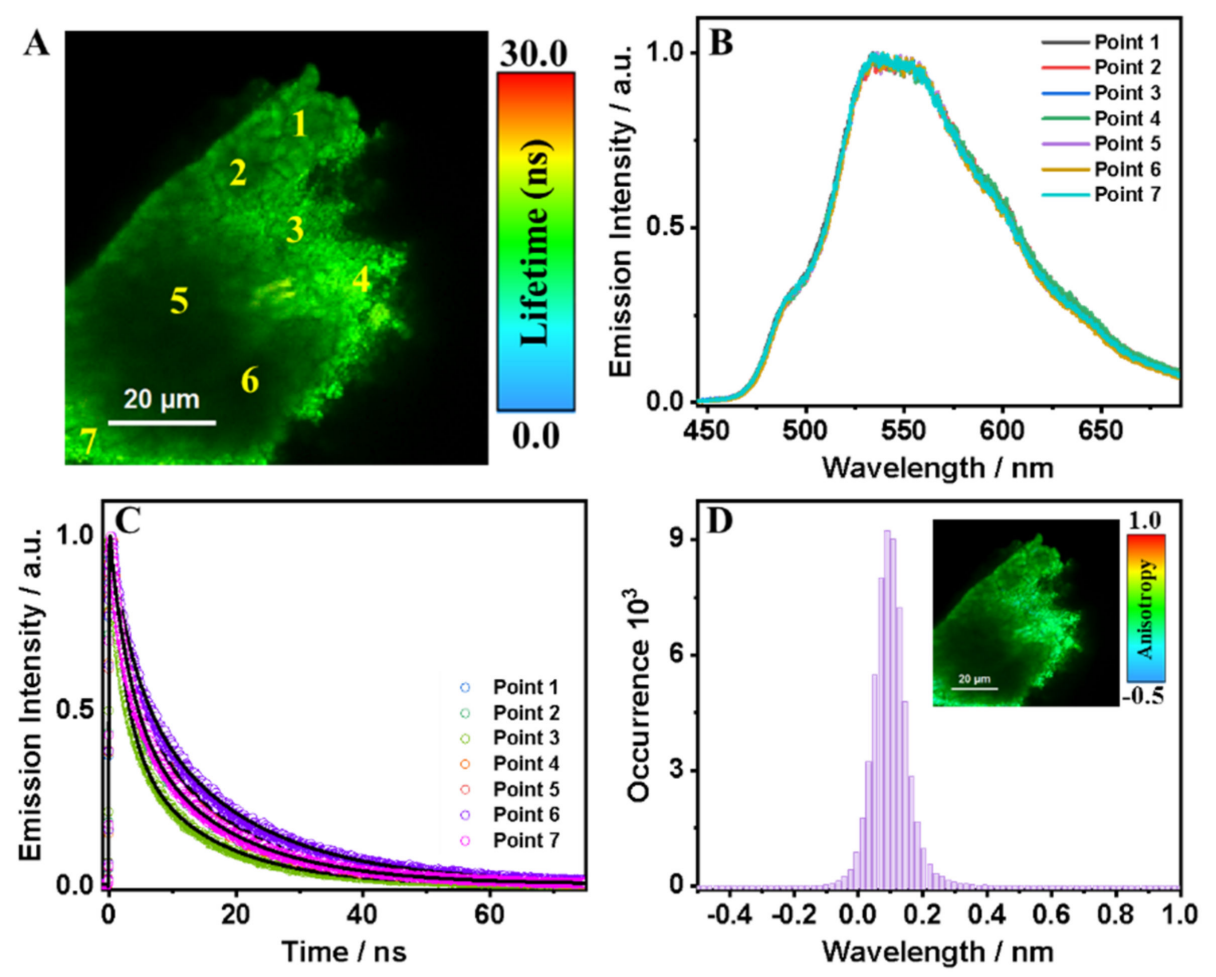
(**A**) FLIM image, (**B**) emission spectra, and (**C**) emission decays at different points for T12F-ester in the solid state. The solid lines are from the best fit using a multiexponential function. (**D**) Histogram of the emission anisotropy of T12F-ester in the solid state. The inset in (**D**) shows the anisotropy distribution image of the material. The excitation wavelength was 390 nm.

To begin with, the FLIM of T12F-ester shows uniform lifetime and intensity distribution, which indicates a homogenous distribution (spatial resolution: 300 nm) of the emitting species (Figure 5A).

This observation is supported by the lack of change in the emission spectra collected at different points in the amorphous solid (Figure 5B). These are characterized by an intensity maximum at 535 nm, and several small shoulders at 492, 597, and 640 nm. The decays are also practically independent of the interrogated position in the material (Figure 5C). A multiexponential fit gives average fluorescence lifetimes of τ_1_ = 3.2 ns (45%) and τ_2_ = 14.0 ns (55%), as shown in Table 4.

Figure S10 and Table S3 show FLIMs of several amorphous solids of T12F-ester, which behave in a similar way. Based on our previous report on T12-ester under comparable conditions (fluorescence lifetime of 5 ns and 20 ns due to ICT and molecular structures interacting through π–π interactions, respectively), we suggest the following scenario [8]. For T12F-COOMe, the 3.2 and 14.0 ns components reflect the emission of molecules that have undergone an ICT reaction and those π–π interacting, respectively. However, we cannot exclude that the short component might reflect the presence of defects as the material is clearly amorphous, opening the door to several relaxation pathways, as has been observed even in crystalline structures of MOFs and HOFs [9,23,31,32,33,34,35,36,37].

To obtain information on the spectral dynamic of T12F-ester crystals, we recorded the emission decays using two different filters: blue detection (450–490 nm) and red detection (520–700 nm) (shaded areas in Figure S11). A multiexponential fit gives average fluorescence lifetimes of τ_1_ = 2.2 ns (53%) and τ_2_ = 14.2 ns (47%) for the blue region and τ_1_ = 2.9 ns (46%) and τ_2_ = 15.1 ns (54%) for the red region, as shown in Table S4. The values of the time components are comparable, but their relative amplitudes differ depending on the spectral range of observation. For the blue-emission decays, the relative amplitude of the shortest lifetime is 53%; for the longest lifetime, it is 47%; while for those collected at the red region, they are 46% and 54%, respectively. Thus, the emission spectra of the two emissive species strongly overlap while having very different lifetimes. The anisotropy distribution derived from the FLIM images of T12F-ester is centered around 0.08 (Figure 5D), very close to zero, which supports that there is no preferential orientation of the molecular dipole moment of the ligands in the amorphous structure, in which the main force originates from π–π interactions between the cores of the T12F-COOMe molecules.

**Figure 6:**
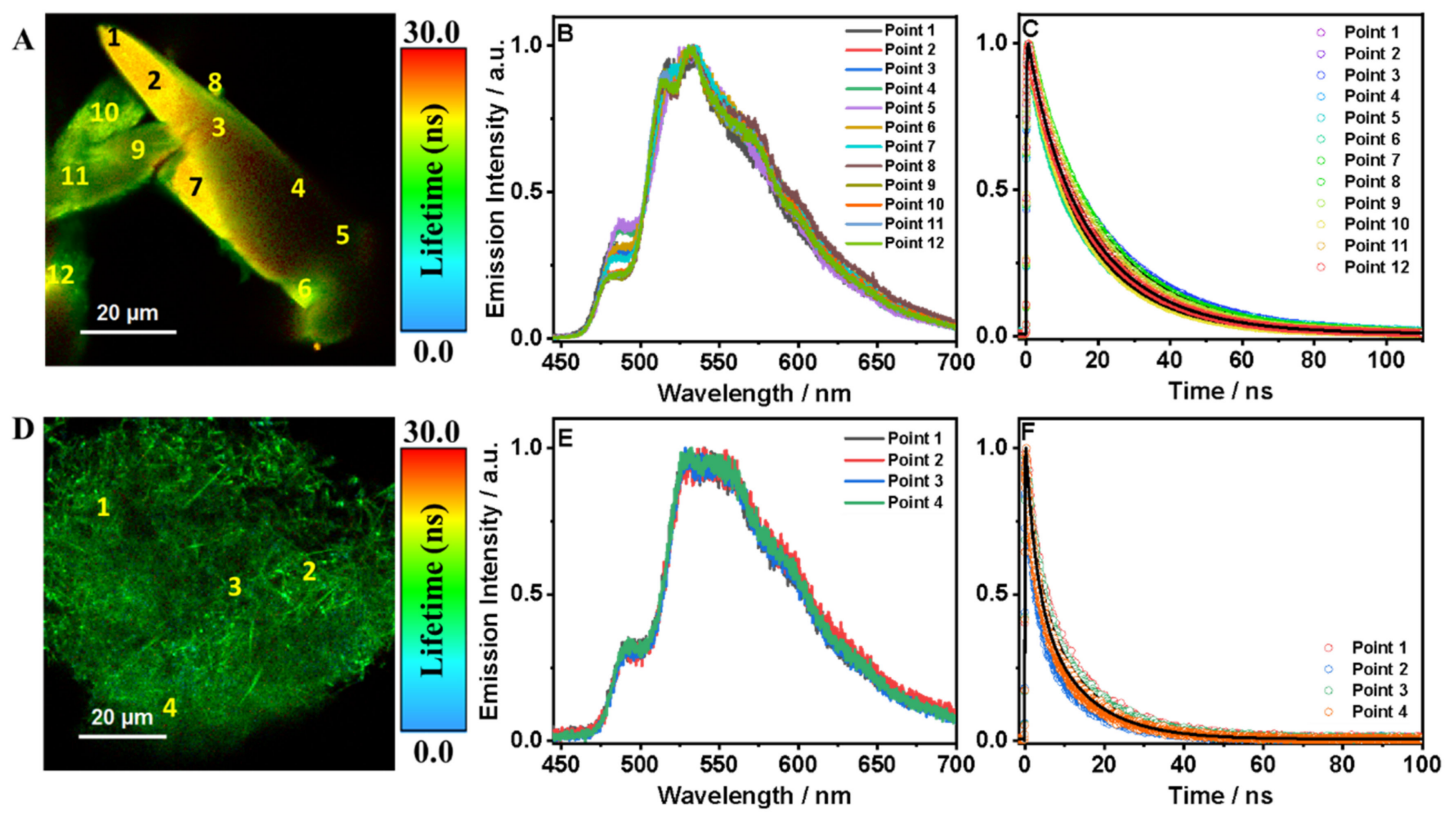
(**A**) FLIM image, (**B**) emission spectra, (**C**) emission decays at different points, (**D**) FLIM images, (**E**) emission spectra and (**F**) emission decays at different points of T12F-1(124TCB) crystal. The solid lines are from the best fit using a multiexponential function. The inset in (**C**,**F**) are the histogram of the emission anisotropy and the anisotropic images of T12F-1(124TCB) in the solid state. The excitation wavelength was 390 nm.

We then examined T12F-1(124TCB) under the fluorescence microscope, where we observed two different kinds of crystalline structures (Figure 6).

Figure 6A displays the large, needle-shaped crystals (LC), while Figure 6D displays much smaller crystals (SC), causing the material to have a cotton-like shape. The color of both FLIM images shows that LCs exhibit a longer fluorescence lifetime than SCs. This behavior has been observed for the HOF based on the parent ligand (T12-COOH) [8]. The recorded emission spectra of LCs observed at different points are practically independent of the focused site, except for a small change in the shoulder intensity at 486 nm (Figure 6B). LCs have a maximum intensity at 532 nm and a vibrational structure with shoulders at 486, 515 and 570 nm. Figure S12 and Table S5 demonstrate more examples that show the same behavior of LCs of T12F-1(124TCB). A multiexponential fit of the emission decays gives average fluorescence lifetimes of τ_1_ = 5.3 ns (18%) and τ_2_ = 21.3 ns (82%), as shown in Table 5.

Based on the discussion of the T12F-ester results, we propose that τ_1_ can be attributed to the lifetime of the charge-transfer species probably located in the regions of chemical or physical defects. We assign τ_2_ to the lifetime of molecular units that have established both a H-bond and π–π interactions to form the crystalline HOF material, as we observed in other HOFs [8,23]. Figure S13C,D show the emission decays collected at blue (450–490 nm) and red (520–700 nm) regions (shaded areas in Figure S13B) for T12F-1(124TCB) crystals (Figure S13A). The fits give time constants of τ_1_ = 4.4 ns (22%) and τ_2_ = 21.4 ns (78%) for the blue zone, and τ_1_ = 4.9 ns (16%) and τ_2_ = 22.0 ns (84%) for the red zone, suggesting that bot species emit in the same spectral range, but of different relaxation pathways (Table S6).

For the SCs of T12F-1(124TCB), the photobehavior is reminiscent of that for T12F-ester, where the shape and position of the emission spectra are independent of the interrogated zone with an intensity maximum at 530 nm and two shoulders at 493 and 591 nm (Figure 6E). Figure S14 and Table S7 show more examples of this kind of behavior. Moreover, the fluorescence lifetimes are shorter than those of LCs of T12F-1(124TCB): τ_1_ = 2.7 ns (47%) and τ_2_ = 12.6 ns (53%) (Table 5). However, they are comparable to the fluorescence lifetimes of T12F-ester (τ_1_ = 3.2 ns (45%) and τ_2_ = 14.0 ns (55%). Considering the spectral and lifetime observations, we suggest that these SCs display a structure that is similar to that of solid T12F-ester, where π–π interactions are the main force behind the crystallization of amorphous solids.

The anisotropy distribution derived from the FLIMs of several LCs and SCs of T12F-1(124TCB) are centered around 0.0 (Figure 6). This is because we could not isolate single crystals to interrogate their emission–anisotropy distribution. However, the anisotropy of the LCs shows a shoulder at 0.25. Moreover, other examples of LCs present values quite different from zero, such as −0.1, 0.1, or even 0.6, suggesting that HOFs display an ordered and crystalline structure, instead of the amorphous small solid or T12F-ester (Figure S15).

Finally, aiming to reveal a photonic application of T12F-1(124TCB) HOF, i.e., in LED technology, we calculated the Commission International de L’Eclairage (CIE) coordinates, which were found to be 0.36, 0.56 (Figure S16). Blue and red emitting molecules may be encapsulated within this HOF to produce a tunable color of light, as we have reported in Zr-based MOF [38]. In a previous report, we suggested the possibility of generating white light using another HOF with similar CIE coordinates (0.42, 0.55) [8]. The procedure will be to mix the emission of the HOF with a blue-emitting dye or LED to generate white light.

## 3. Materials and Methods

^1^H and ^13^C NMR spectra (Figures S17–S24) were recorded using a JEOL or Bruker spectrometer (400 MHz for ^1^H and 100 MHz for ^13^C). Mass spectrum data were obtained from a JEOL JMS-700 instrument or Autoflex III, Bruker. For single crystal X-ray crystallographic analysis: diffraction data were collected at 100 K on a DECTRIS EIGER X 1M diffractometer with Si (111) mono-chromated synchrotron radiation (λ = 0.81089 Å) at BL40XU (SPring-8). SHELXT was used for the structure solution of the crystal [39]. All calculations were performed with the observed reflections [I > 2σ(I)] with the program CrystalStructure [40]. Refinement was performed using SHELXL [39,41].

*N,N*-dimethylformamide (DMF, 99.8%, spectroscopic grade) and 1,2,4-trichlorobenzene (1,2,4-TCB, 99.8%, spectroscopic grade) used for the spectroscopic measurements were purchased from Sigma-Aldrich (Madrid, Spain) and used as received. Steady-state UV-visible absorption and emission spectra were recorded using JASCO V-670 (Madrid, Spain) and FluoroMax-4 (Jobin-Yvon, Madrid, Spain) spectrophotometers, respectively. The fluorescence quantum yields (ϕ) were measured using quinine sulphate in a 0.1 N H_2_SO_4_ solution as a reference (ϕ = 0.51 at 293 K) [42].

Picosecond (ps) emission decays were measured using a time-correlated single photon counting (TCSPC) system [43]. The nanosecond (ns) flash photolysis setup has been described previously [44]. The fluorescence lifetime imaging (FLIM) measurements were performed on a MicroTime 200 confocal microscope (PicoQuant, Berlin, Germany). As an excitation source, we used a diode laser with an excitation wavelength of 390 nm (40 ps full width at half-maximum of intensity) [14,45,46].

## 4. Conclusions

Herein, we reported on the synthesis, characterization, and spectroscopic studies of a new dehydrobenzoannulene (DBA) derivative, T12F-COOH, and its methylated ester, T12F-COOMe, in solution and in the solid state. T12F-COOH forms a 2D-MOF (a porous sheet that is hexagonally networked via H-bonds between the carboxylic groups). The slip-stacked sheets of the hydrogen-bonded organic framework (HOF) exhibit two kinds of voids with smaller triangular and larger hexagonal apertures. The interlayers distance (~4.3 Å) is clearly longer than that of a typical π–π interaction. The void channels of the HOF with an aperture size of 17 Å × 8 Å are formed along the a-axis, accommodating solvent molecules. The inductive effect of the fluorine atoms in the DBA molecular units largely increases the emission quantum yield (40% for T12F-COOMe and 33% for T12F-COOH in DMF solution) when compared to the parent compounds. The time-resolved photobehavior of both T12F-COOMe and T12F-COOH in DMF solution is governed by the emission from a locally excited state (S1, ~0.4 ns), a charge transfer state (S1(CT), ~2 ns) generated by an ultrafast charge transfer process (<15 ps), and from an emissive triplet state (T1, ~20 ns). Additionally, we observed a non-radiative triplet state with a charge-transfer character (T1(CT), τ = 0.75 µs). Picosecond time-resolved FLIM experiments on the HOF show that the fluorescence lifetimes of the LC structures are almost twice as long as those of amorphous SCs and the T12F-ester solid, showing the relevance of the H-bonds in the photodynamics of the HOF. These new results provide the background for further developments of DBAs-based HOFs for possible applications in lighting, sensing, or imaging.

## Figures and Tables

**Table 1:** ^a^ Dihedral angle (°) between the dehydrobenzoannulene (DBA) core and the peripheral phenylene group. ^b^ Dihedral angle (°) between the phenylene and carboxylic groups. ^c^ Dihedral angle (°) between the DBA core and carboxylic groups.

Groups	Arm A	Arm B	Arm C
DA(dba-phe) ^a^	+50.97	−46.06	−50.57
DA(phe-cbx) ^b^	+32.06	−40.67	−33.54
DA(dba-cbx) ^c^	+83.02	−86.11	−84.09

**Table 2:** Values of time constants (τ_i_), normalized (to 100) pre-exponential factors (a_i_), and contributions (c_i_ = τ_i_ × a_i_/∑(τ_i_ × a_i_)) in the signal obtained from a global multiexponential fit for T12F-COOMe and T12F-COOH in DMF solution, and upon excitation at 390 nm. The observation wavelengths are indicated.

Sample	λ_obs_ (nm)	τ_1_ (ps) ± 50 ps	A_1_	c_1_	τ_2_ (ns) ± 0.2 ns	A_2_	c_2_	τ_3_ (ns) ± 2.0 ns	A_3_	c_3_
**T12F-COOMe**	450 460 470 480 500 525 550 575 600 650	470	65 41 23 16 1 - - - - -	11 2 1 1 1 - - - - -	2.5	28 24 16 15 9 7 6 5 5 5	25 7 3 2 1 1 1 1 1 1	23.1	7 35 62 69 90 93 94 95 95 95	64 91 96 97 98 99 99 99 99 99
**T12F-COOH**	450 460 470 480 500 525 550 575 600 650	400	64 35 22 - - - - - - -	6 1 1 - - - - - - -	2.0	24 17 10 12 11 10 10 10 10 10	15 3 1 1 1 1 1 1 1 1	21.4	12 48 68 88 89 90 90 90 90 90	79 96 98 99 99 99 99 99 99 99

**Table 3:** Values of time constants (τ_i_) and normalized (to 100) pre-exponential factors (a_i_) obtained from the best fits of the transient absorption decays of T12F-COOMe and T12F-COOH in DMF solution upon excitation at 355 nm, and probing wavelengths as indicated. * The signal is negative in sign, reflecting an emissive process. The errors in the time constant values are about 15%.

Sample	λ_obs_ (nm)	Conditions	τ_1_ (ns)	a_1_	τ_2_ (µs)	a_2_
**T12F-COOMe**	410	Air	35	70	0.76	30
		O_2_	15	95	0.11	5
	520	Air	30 *	100 *	0.79	100
		O_2_	10 *	100 *	0.12	100
	700	Air	10 *	100 *	0.72	100
		O_2_	8 *	100 *	0.15	100
**T12F-COOH**	410	Air	35	67	0.76	33
		O_2_	16	97	0.10	3
	520	Air	28 *	100 *	0.77	100
		O_2_	20 *	100 *	0.18	100
	700	Air	10 *	100 *	0.73	10
		O_2_	13 *	100 *	0.12	100

**Table 4:** Values of time constants (τ_i_) and normalized (to 100) pre-exponential factors (a_i_) obtained from the fit of the emission decays collected at the selected points of T12F-ester (Figure 5). The errors in the time constant values are about 20%.

Sample	Point	τ_1_ (ns)	a_1_	τ_2_ (ns)	a_2_
**T12F-ester**	1 2 3 4 5 6 7	3.3 3.1 3.0 3.3 3.3 3.3 3.2	44 49 42 49 44 38 50	14.1 14.2 13.5 14.1 14.2 14.2 13.8	56 51 58 51 56 62 20
	Average	3.2	45	14.0	55

**Table 5:** Values of time constants (τ_i_) and normalized (to 100) pre-exponential factors (a_i_) obtained from the best fit of the emission decays collected at selected points of T12F-1(124TCB), as indicated in Figure 6. The errors in the time constant values are about 20%.

Sample	Point	τ_1_ (ns)	a_1_	τ_2_ (ns)	a_2_
**T12F-1(124TCB)** of Figure 6A	1 2 3 4 5 6 7 8 9 10 11 12	5.0 5.1 5.1 5.0 5.2 5.2 5.5 5.5 5.2 5.5 5.5 5.5	19 20 15 15 15 27 22 15 10 25 10 27	22.0 21.9 22.2 21.5 21.3 21.0 21.0 21.1 21.1 21.0 21.0 21.0	81 80 85 85 85 73 78 85 90 75 90 73
	Average	5.3	18	21.3	82
**T12F-1(124TCB)** of Figure 6D	1 2 3 4	2.7 2.6 2.8 2.7	40 57 41 49	12.7 13.0 12.5 12.3	60 43 59 51
	Average	2.7	47	12.6	53

## Data Availability

Not applicable.

## Supplementary Materials

The following are available online at https://www.mdpi.com/article/10.3390/ijms22094803/s1, Scheme S1: Synthesis of the coumponds, Figure S1: Predicted structures of T12F-COOMe and T12F-COOH molecules, Figure S2: Stacking manner of the adjacent layers in T12F-1(124TCB), Figure S3: Visualization of the contact surfaces of the channel of T12F-1(124TCB), Figure S4: Selected framework of T12F-1(124TCB) containing solvent molecules, Figures S5–S8: Absorption and emission spectra and decays of T12F-COOMe and T12F-COOH in DMF solutions, Figure S9: Microsecond transient absorption spectra and decays of T12F-COOMe and T12F-COOH in DMF solutions, Figures S10–S14: Emission spectra and decays of T12-ester FLIM of different crystals, Figure S15: Emission anisotropy histogram of T12F-1(124TCB) crystals, Figure S16: Emission spectra and CIE coordinates of T12F-1(124TCB), Figures S17–S24: Proton NMR spectra of the synthesized intermediates and final coumpounds. Table S1; Crystallographic data of T12F-1(124TCB), Table S2: Photophysical data of T12F-COOMe and T12F-COOH in DMF solutions, Tables S3–S7: Emission lifetime values of the ester-sample FLIM under different observation conditions.

## References

[1] Luo J., Wang J.W., Zhang J.H., Lai S., Zhong D.C. (2018). Hydrogen-bonded organic frameworks: Design, structures and potential applications. CrystEngComm.

[2] Lin R.B., He Y., Li P., Wang H., Zhou W., Chen B. (2019). Multifunctional porous hydrogen-bonded organic framework materials. Chem. Soc. Rev..

[3] Hisaki I., Xin C., Takahashi K., Nakamura T. (2019). Designing Hydrogen-Bonded Organic Frameworks (HOFs) with Permanent Porosity. Angew. Chem. Int. Ed..

[4] Yang W., Yang F., Hu T.L., King S.C., Wang H., Wu H., Zhou W., Li J.R., Arman H.D., Chen B. (2016). Microporous Diaminotriazine-Decorated Porphyrin-Based Hydrogen-Bonded Organic Framework: Permanent Porosity and Proton Conduction. Cryst. Growth Des..

[5] Hu F., Liu C., Wu M., Pang J., Jiang F., Yuan D., Hong M. (2017). An Ultrastable and Easily Regenerated Hydrogen-Bonded Organic Molecular Framework with Permanent Porosity. Angew. Chem. Int. Ed..

[6] Karmakar A., Illathvalappil R., Anothumakkool B., Sen A., Samanta P., Desai A.V., Kurungot S., Ghosh S.K. (2016). Hydrogen-Bonded Organic Frameworks (HOFs): A New Class of Porous Crystalline Proton-Conducting Materials. Angew. Chem. Int. Ed..

[7] Sun Z., Li Y., Chen L., Jing X., Xie Z. (2015). Fluorescent Hydrogen-Bonded Organic Framework for Sensing of Aromatic Compounds. Cryst. Growth Des..

[8] Gomez E., Gutiérrez M., Cohen B., Hisaki I., Douhal A. (2018). Single crystal fluorescence behavior of a new Hof material: A potential candidate for a new LED. J. Mater. Chem. C.

[9] Hisaki I., Suzuki Y., Gomez E., Ji Q., Tohnai N., Nakamura T., Douhal A. (2019). Acid Responsive Hydrogen-Bonded Organic Frameworks. J. Am. Chem. Soc..

[10] Gomez E., Suzuki Y., Hisaki I., Moreno M., Douhal A. (2019). Spectroscopy and dynamics of a HOF and its molecular units: Remarkable vapor acid sensing. J. Mater. Chem. C.

[11] Marchesan S., Prato M. (2013). Nanomaterials for (Nano)medicine. ACS Med. Chem. Lett..

[12] Wen A.M., Steinmetz N.F. (2016). Design of virus-based nanomaterials for medicine, biotechnology, and energy. Chem. Soc. Rev..

[13] Yin Q., Zhao P., Sa R.J., Chen G.C., Lü J., Liu T.F., Cao R. (2018). An Ultra-Robust and Crystalline Redeemable Hydrogen-Bonded Organic Framework for Synergistic Chemo-Photodynamic Therapy. Angew. Chem. Int. Ed..

[14] Hisaki I., Ikenaka N., Gomez E., Cohen B., Tohnai N., Douhal A. (2017). Hexaazatriphenylene-Based Hydrogen-Bonded Organic Framework with Permanent Porosity and Single-Crystallinity. Chem. Eur. J..

[15] Zhou H., Ye Q., Wu X., Song J., Cho C.M., Zong Y., Tang B.Z., Hor T.S., Yeow E.K., Xu J. (2015). A thermally stable and reversible microporous hydrogen-bonded organic framework: Aggregation induced emission and metal ion-sensing properties. J. Mater. Chem. C.

[16] Wang H., Bao Z., Wu H., Lin R.B., Zhou W., Hu T.L., Li B., Zhao J.C., Chen B. (2017). Two solvent-induced porous hydrogen-bonded organic frameworks: Solvent effects on structures and functionalities. Chem. Commun..

[17] Liu T., Wang B., He R., Arman H., Schanze K.S., Xiang S., Li D., Chen B. (2020). A novel hydrogen-bonded organic framework for the sensing of two representative organic arsenics. Can. J. Chem..

[18] Tahara K., Yamamoto Y., Gross D.E., Kozuma H., Arikuma Y., Ohta K., Koizumi Y., Gao Y., Shimizu Y., Seki S. (2013). Syntheses and Properties of Graphyne Fragments: Trigonally Expanded Dehydrobenzo [12]annulenes. Chem. Eur. J..

[19] Yoshimura T., Inaba A., Sonoda M., Tahara K., Tobe Y., Williams R.V. (2006). Synthesis and Properties of Trefoil-Shaped Tris(hexadehydrotribenzo[12]annulene) and Tris(tetradehydrotribenzo[12]annulene). Org. Lett..

[20] Johnson C.A., Lu Y., Haley M.M. (2007). Carbon Networks Based on Benzocyclynes. 6. Synthesis of Graphyne Substructures via Directed Alkyne Metathesis. Org. Lett..

[21] Hisaki I., Nakagawa S., Tohnai N., Miyata M. (2015). A C3-Symmetric Macrocycle-Based, Hydrogen-Bonded, Multiporous Hexagonal Network as a Motif of Porous Molecular Crystals. Angew. Chem. Int. Ed..

[22] Hisaki I., Nakagawa S., Ikenaka N., Imamura Y., Katouda M., Tashiro M., Tsuchida H., Ogoshi T., Sato H., Tohnai N. (2016). A Series of Layered Assemblies of Hydrogen-Bonded, Hexagonal Networks of C3-Symmetric π-Conjugated Molecules: A Potential Motif of Porous Organic Materials. J. Am. Chem. Soc..

[23] Gomez E., di Nunzio M.R., Moreno M., Hisaki I., Douhal A. (2020). Shape-Persistent Phenylene-Ethynylene Macrocycles Spectroscopy and Dynamics: From Molecules to the Hydrogen-Bonded Organic Framework Material. J. Phys. Chem. C.

[24] Hisaki I., Sakamoto Y., Shigemitsu H., Tohnai N., Miyata M., Seki S., Saeki A., Tagawa S. (2008). Superstructure-Dependent Optical and Electrical Properties of an Unusual Face-to-Face, π-Stacked, One-Dimensional Assembly of Dehydrobenzo[12]annulene in the Crystalline State. Chem. Eur. J..

[25] Gomez E., Gutierrez M., Moreno M., Hisaki I., Nakagawa S., Douhal A. (2018). Spectroscopy and dynamics of dehydrobenzo[12]annulene derivatives possessing peripheral carboxyphenyl groups: Theory and experiment. Phys. Chem. Chem. Phys..

[26] Alarcos N., Gutiérrez M., Liras M., Sánchez F., Douhal A. (2015). From intra- to inter-molecular hydrogen bonds with the surroundings: Steady-state and time-resolved behaviours. Photochem. Photobiol. Sci..

[27] Lewis F.D., Kalgutkar R.S., Yang J.S. (1999). The Photochemistry of trans-ortho-, -meta-, and -para-Aminostilbenes. J. Am. Chem. Soc..

[28] Lewis F.D., Kalgutkar R.S. (2001). The Photochemistry of cis-ortho-, meta-, and para-Aminostilbenes. J. Phys. Chem. A.

[29] Lakowicz J.R. (2007). Principles of Fluorescence Spectroscopy.

[30] Valeur B. (2009). Molecular Fluorescence. Digit. Encycl. Appl. Phys..

[31] Alarcos N., Cohen B., Ziółek M., Douhal A. (2017). Photochemistry and Photophysics in Silica-Based Materials: Ultrafast and Single Molecule Spectroscopy Observation. Chem. Rev..

[32] Gutiérrez M., Martin C., Kennes K., Hofkens J., Van der Auweraer M., Sánchez F., Douhal A. (2018). New OLEDs Based on Zirconium Metal-Organic Framework. Adv. Opt. Mater..

[33] Reshchikov M.A., Morkoç H. (2005). Luminescence properties of defects in GaN. J. Appl. Phys..

[34] Xiang W., Zhang Y., Chen Y., Liu C.J., Tu X. (2020). Synthesis, characterization and application of defective metal–organic frameworks: Current status and perspectives. J. Mater. Chem. A.

[35] Fang Z., Bueken B., De Vos D.E., Fischer R.A. (2015). Defect-Engineered Metal–Organic Frameworks. Angew. Chem. Int. Ed..

[36] Ameloot R., Vermoortele F., Hofkens J., De Schryver F.C., De Vos D.E., Roeffaers M.B. (2013). Three-Dimensional Visualization of Defects Formed during the Synthesis of Metal–Organic Frameworks: A Fluorescence Microscopy Study. Angew. Chem. Int. Ed..

[37] Di Nunzio M.R., Caballero-Mancebo E., Cohen B., Douhal A. (2020). Photodynamical behaviour of MOFs and related composites: Relevance to emerging photon-based science and applications. J. Photochem. Photobiol. C.

[38] Gutierrez M., Sanchez F., Douhal A. (2015). Efficient multicolor and white light emission from Zr-based MOF composites: Spectral and dynamic properties. J. Mater. Chem. C.

[39] Sheldrick G. (2015). SHELXT-Integrated space-group and crystal-structure determination. Acta Crystallogr. Sect. A.

[40] Rigaku (2018). Crystal Structure.

[41] Sheldrick G. (2008). A short history of SHELX. Acta Crystallogr. Sect. A.

[42] Velapoldi R.A., Mielenz K.D. (1981). A Fluorescence Standard Reference Material-Quinine Sulfate Dihydrate. Appl. Opt..

[43] Organero J.A., Tormo L., Douhal A. (2002). Caging Ultrafast Proton Transfer and Twisting Motion of 1-Hydroxy-2-Acetonaphthone. Chem. Phys. Lett..

[44] Randino C., Ziolek M., Gelabert R., Organero J.A., Gil M., Moreno M., Lluch J.M., Douhal A. (2011). Photo-deactivation pathways of a double H-bonded photochromic Schiff base investigated by combined theoretical calculations and experimental time-resolved studies. Phys. Chem. Chem. Phys..

[45] Angiolini L., Valetti S., Cohen B., Feiler A., Douhal A. (2018). Fluorescence imaging of antibiotic clofazimine encapsulated within mesoporous silica particle carriers: Relevance to drug delivery and the effect on its release kinetics. Phys. Chem. Chem. Phys..

[46] Cohen B., Martin C., Iyer S.K., Wiesner U., Douhal A. (2012). Single Dye Molecule Behavior in Fluorescent Core–Shell Silica Nanoparticles. Chem. Mater..

