# Supplementary material for: Synthesis and Photobehavior of a New Dehydrobenzoannulene-Based HOF with Fluorine Atoms: From Solution to Single Crystals Observation
Source: Int J Mol Sci. 2021 Apr 30;22(9):4803. doi: 10.3390/ijms22094803 (PMC8124357; doi:10.3390/ijms22094803)
Supplement: Supplementary file 1 [file ijms-22-04803-s001.zip › ijms-1167167-supplementary.pdf]

International Journal of
Molecular Sciences

MDPI

Supplementary Material

Synthesis and Photobehavior of A New Dehydrobenzoannulene-Based HOF with Fluorine Atoms: From Solution to Single Crystals Observation

Eduardo Gomez [1], Ichiro Hisaki [2,*] and Abderrazzak Douhal [1,*]

[1] Physical Chemistry Department, Faculty of Environmental Sciences and Biochemistry, and INAMOL, University of Castilla-La ManchaDepartamento de Química Física, Facultad de Ciencias Ambientales y Bioquímica, and INAMOL, Universidad de Castilla-La Mancha, Avenida Carlos III, S.N., 45071 Toledo, Spain; eduardo.gomez@uclm.es

[2] Division of Chemistry, Graduate School of Engineering Science, Osaka University, 1-3 Machikaneyama, Toyonaka, Osaka 560-8531, Japan

* Correspondence: hisaki@chem.es.osaka-u.ac.jp and abderrazzak.douhal@uclm.es

1. General

2. Synthesis of T12F-COOMe, T12F-COOH and the corresponding material and HOF.

3. Crystallography

4. Optical properties of the compounds

5. NMR spectra of synthesized compounds

1. General

1.1. X-Ray Diffraction Measurements

For single crystal X-ray measurement and analysis, diffraction data of T12F-1(124TCB) were collected at Spring-8 (BL40XU) with synchrotron radiation (λ = 0.81106 Å). Cell refinements were performed with the RapidAuto software. SHELXT was used for the structure solution of the crystal[1]. All calculations were performed with the observed reflections [$I > 2\sigma(I)$] with the program CrystalStructure. Structural refinement was performed by SHELXL[2]. All non-hydrogen atoms were refined with anisotropic displacement parameters, and hydrogen atoms were placed in idealized positions and refined as rigid atoms with the relative isotropic displacement parameters. The SQUEEZE function equipped in the PLATON program was used to treat severely disordered solvent molecules in voids[3,4].

1.1.1. Picosecond-Time Resolved Fluorescence Experiments Using a TCSPC

Picosecond (ps) emission decays were measured using a time-correlated single photon counting (TCSPC) system[5]. For excitation at 390 nm, the samples were excited by a 40 ps pulsed diode laser (<5 mW, 40 MHz repetition rate) and the instrument response function (IRF) was ~70 ps. The IRF of the system for solution samples was measured using a standard LUDOX (Sigma-Aldrich) suspension in a 1 cm cell, while for solid state samples, the IRF was measured using a portion of the reflected laser. The decays were deconvoluted and fitted to a multiexponential function using the FLUOFIT (version: 4.6.6.0) package (PicoQuant), allowing single and global fits. The quality of the fit was estimated by χ^2, which was always below 1.2, and the residuals distribution.

1.1.2. The Nanosecond (ns) Flash Photolysis Setup

As previously described,[6] this set up consists of an LKS.60 laser flash photolysis spectrometer (Applied Photophysics) and a Vibrant (HE) 355 II laser (Oppotek). To excite the sample, we used the third harmonic output (355 nm). The pump fluence was ~80 $mJcm^{-2}$. As a probe light, we used the output of a 150 W Xenon arc lamp. The light transmitted through the sample was then dispersed by a monochromator and detected by a visible photomultiplier (Applied Photophysics R928) coupled to a digital oscilloscope (Agilent Infiniium DS08064A, 600 MHz, 4 GSa s1). The measured IRF of the system is ~8 ns. All experiments were performed at 293 K and in a 1 cm cell.

1.1.3. Time-Resolved FLIM Experiments

The fluorescence lifetime imaging (FLIM) measurements were performed on a MicroTime 200 confocal microscope (PicoQuant). As an excitation source, we used a diode laser with an excitation wavelength of 390 nm (40 ps full width at half-maximum of intensity). Briefly, it consisted of an inverse Olympus IX 71 microscope equipped with a water immersion objective (60× NA1.2, Olympus) and a 2D piezo scanner (Physik Instrumente). The emitted light was then focused on a 50 μm pinhole and later collimated to two independent single photon avalanche photodiodes (Micro-Photon Devices) for time-resolved measurements. The emission spectra were collected through a Shamrock SR-303i (Andor Technology) imaging spectrograph and detected by an Andor Newton EMCCD camera (Andor Technology). For the anisotropy measurements, the light was passed through a polarizing beam-splitter that allowed for the simultaneous detection of the parallel and perpendicular parts of the emission. A G-factor of 1.2, accounting for differences in the detection sensitivity for both polarizations in the setup, was used in the calculation of the anisotropy. To calculate the histogram of the anisotropy, we used the SymPhoTime Analysis program (PicoQuant), which calculates the static anisotropy of an image[7]. The limits for the anisotropy are -0.5 to 1.0 (-0.5 corresponds to perpendicular orientation and 1.0 to parallel)[8]. The emission spectra were collected using long pass filters (HQ430LP

Chroma), while the emission decays were collected using different types of filters depending on the interrogated region. We used FF01-470-28-25 Chroma and HQ520/40LP Chroma filters.

2. Syntheis of T12F-COOMe, T12F-COOH, and the Corresponding Material and HOF

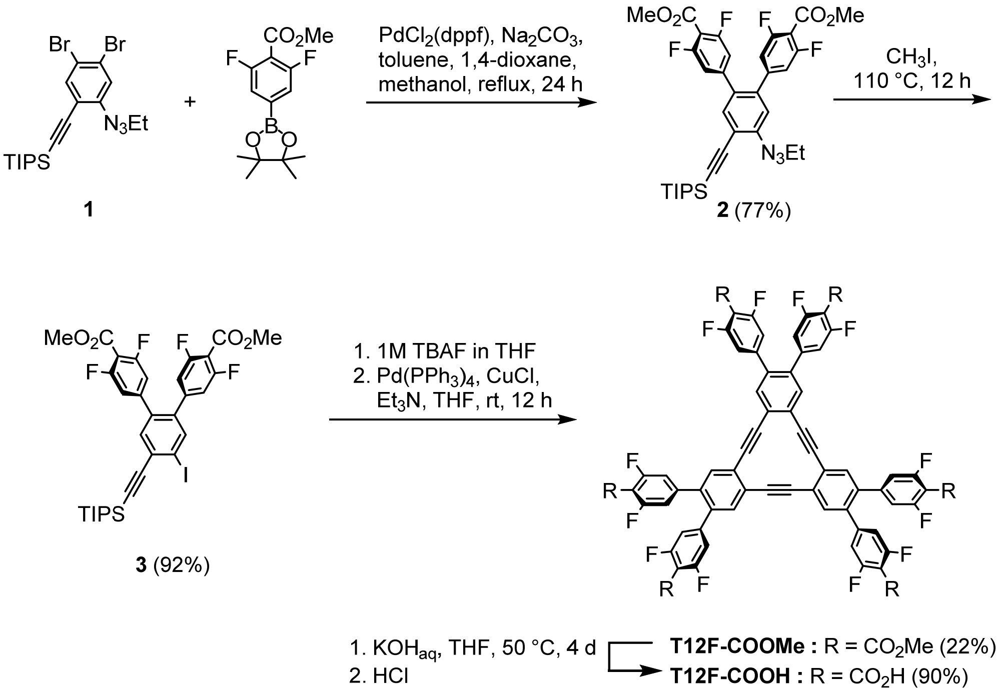

Scheme S1. Synthesis of T12F-COOMe and T12F-COOH.

***o*-Terphenyl derivative 2**: A mixture of ((2-azido-4,5-dibromophenyl)ethynyl)triisopropylsilane (**1**)[9] (394 mg, 0.764 mmol), 3,5-difluoro-4-methoxycarbonylphenylboronic acid pinacolyl ester (500 mg, 1.68 mmol), $PdCl_2$ (dppf) (135 mg, 0.184 mmol), and Na_2CO_3 (250 mg, 2.36 mmol) in deoxygenated toluene (10 mL), 1,4-dioxane (5 mL), and methanol (5 mL) was stirred for 24 h under reflux conditions. The reaction mixture was extracted with chloroform, washed with water and brine, and dried over anhydrous Na_2SO_4. The product was purified by column chromatography (silica gel, hexane:EtOAc = 98:2) to give **2** (408 mg, 0.59 mmol, 77%) as a yellow solid.

2: MP: 193–196 °C. ^{1}H NMR (400 MHz, $CDCl_3$): δ 7.48 (s, 1H), 7.46 (s, 1H), 6.74 (m, 4H), 3.94 (s, 6H), 3.84 (*t*, 3H, *J* = 8 Hz), 1.34 (*t*, 3H, *J* = 8 Hz), 1.24 (*t*, 3H, *J* = 8 Hz) and 1.14 (s, 21H) ppm. ^{13}C NMR (100 MHz, $CDCl_3$): 161.75, 159.12, 152.66, 146.03, 145.46, 137.42, 135.80, 133.00. 119.18, 118.69, 113.29, 113.05, 110.86, 110.59, 109.34, 109.11, 103.86, 96.65, 52.76, 49.27, 41.68, 18.71, 14.45, 11.33 and 11.05 ppm. HR-MS (FAB) m/z calc. for $[M+H]^+$ $C_{37}H_{44}F_4N_3O_4Si$: 698.3037; found: 698.30317.

Iodobenzene derivative 3: To a solution of **2** (515 mg, 0.74 mmol) in CH_3I (40 mL), iodine (43 mg) was added, sealed in a pressure bottle, and stirred for 12 h at 110 °C. After the solvent was removed in vacuo, the product was extracted by dichloromethane, washed with 5% $Na_2S_2O_3$ aqueous solution and brine, and dried over anhydrous Na_2SO_4. The product was purified by column chromatography (silica gel) to give **3** (477 mg, 0.65 mmol, 92%) as a yellow solid.

3: MP: 153–155 °C. ^{1}H NMR (400 MHz, $CDCl_3$): δ 7.88 (s, 1H), 7.45 (s, 1H), 6.73 (*t*, 4H, *J* = 8 Hz), 3.96 (s, 6H) and 1.18 (s, 21H) ppm. ^{13}C NMR (100 MHz, CDCl3): 161.82, 161.47, 159.29, 159.22, 144.17, 143.59, 140.31, 137.77, 137.02, 134.32, 131.30, 113.18, 112.96, 109.97, 106.58, 101.46, 96.41, 52.86, 18.70 and 11.26 ppm. HR-MS (FAB) m/z calc. for $[M+Na]^+$ $C_{33}H_{33}F_4IO_4NaSi$: 747.1027; found: 747.1021.

Cyclic trimer T12F-COOMe: To a solution of **3** (525 mg, 0.724 mmol) in THF (50 mL), a 1M solution of tetrabutylammonium fluoride in THF (0.72 mL) was added and stirred for 2 h at room temperature. After this time, the reaction was quenched by water, extracted by chloroform, washed with water and brine, dried over anhydrous Na_2SO_4, and purified by column chromatography (silica gel, chloroform). The purified compound (104 mg, 0.189 mmol) was dissolved in THF (5 ml) and triethylamine (5 ml) and CuCl (15 mg, 0.012 mmol) and $(PPh_3)_4Pd$ (3.5 mg, 0.018 mmol) were added to the solution, which was then stirred at room temperature for 12 h under nitrogen atmosphere. The reaction mixture was concentrated, extracted with chloroform, washed with water and brine, and dried over anhydrous Na_2SO_4. The obtained solid was purified by HPLC to give T12F-COOMe (16.5 mg, 0.012 mmol, 22%) as a yellow solid.

T12F-COOMe: MP: 210 °C (decomp.). ^{1}H NMR (400 MHz, $CDCl_3$): δ 7.43 (s, 6H), 6.76 (*d*, 12H, *J* = 8 Hz) and 3.96 (s, 18H) ppm. ^{13}C NMR (150 MHz, $CDCl_3$): 161.85, 161.44, 159.32, 143.85, 137.91, 134.10, 127.08, 113.13, 112.87, 110.21, 93.37, 52.93 ppm. HR-MS (FAB) m/z calc. for $[M+Na]^+$ $C_{72}H_{36}F_{12}O_{11}Na$: 1343.1913; found: 1343.1907.

T12F-COOH: To a solution of T12F-COOMe (48 mg, 0.036 mol) in THF (25 mL), 10% KOH aqueous solution (10 mL) was added and stirred at 50 °C for 4 days. The THF was evaporated in vacuo, and to the aqueous phase, 6M HCl was added dropwise until the solution became acidic. Finally, the obtained precipitated was filtered and washed with water to yield **T12F-COOH** (39 mg, 0.031 mmol, 90%) as a yellow solid.

T12F-COOH: MP: 255 °C (decomp.). ^{1}H NMR (400 MHz, DMSO-*d*$_6$): δ 7.61 (s, 6H), 7.03 (d, 12H, *J* = 8 Hz) and 3.35 (s) ppm. ^{13}C NMR (100 MHz, DMSO-*d*$_6$): 161.86, 160.03, 157.58, 157.49, 142.67, 138.24, 134.04, 125.83, 113.48, 113.23, 93.17 ppm. HR-MS (FAB) m/z calc. for $[M]^-$ $C_{66}H_{23}F_{12}O_{12}$: 1235.0998; found: 1235.0992.

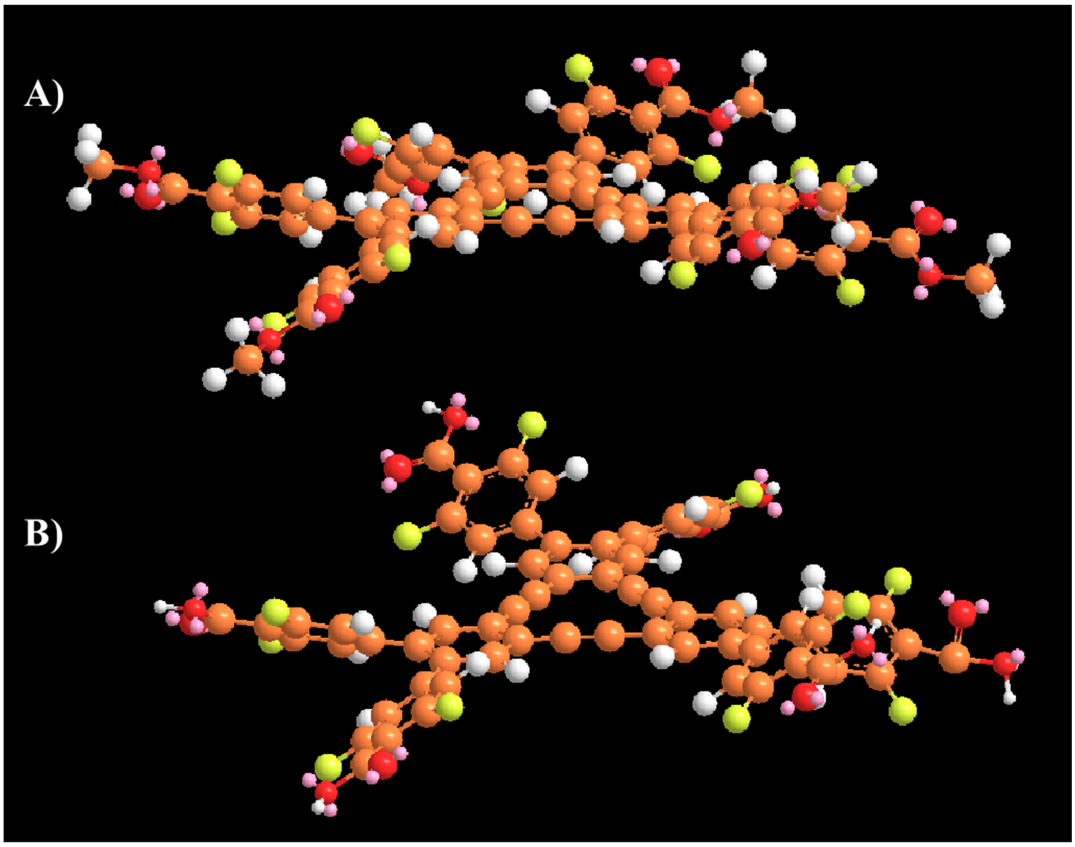

Figure S1. Predicted (ChemBio3D ultra 14.0 (PerkinElmer), MM2) most stable conformation of (**A**) T12F-COOMe and (**B**) T12F-COOH in the gas phase.

3. Crystallography

Table S1. Crystal data of T12F-1(124TCB).

Copyright: © 2021 by the authors. Licensee MDPI, Basel, Switzerland. This article is an open access article distributed under the terms and conditions of the Creative Commons Attribution (CC BY) license (http://creativecommons.org/licenses/by/4.0/).

	**T12F-1(124TCB)**
System	Monoclinic
Space group	*I*2/*a* (no. 15)
Fourmula	$C_{72}H_{27}Cl_3F_{12}O_{12}$
Fourmula weight	1418.29
a / Å	13.6283(4)
b / Å	43.2387(10)
c / Å	17.0639(5)
α / °	90
β / °	109.279(2)
γ / °	90
V (Å^3)	9491.4(4)
Z	4
D / g cm^3	0.993
Crystal size (mm)	0.01 × 0.005 × 0.002
crystal color	Yellow
R1 ($I > 2.0\sigma(I)$)	0.1161
wR2 (all)	0.3766
Completeness	1.00
GOF	1.254
λ / Å	0.81134 (synchrotron)
T / K	100
CCDC no.	2051621

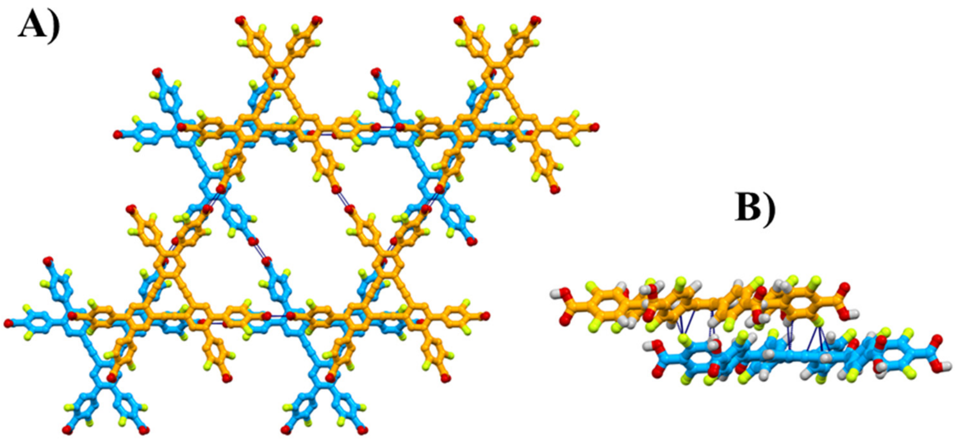

Figure S2. Stacking manner of the adjacent layers in T12F-1(124TCB). (**A**) Rhombic motifs with large overlap of the framework. (**B**) Interlayer contacts between the peripheral difluorophenylene and the central annulene moieties, where dark blue lines indicate short contacts between H or F atoms in the phenylene rings and carbon atoms in the annulene core.

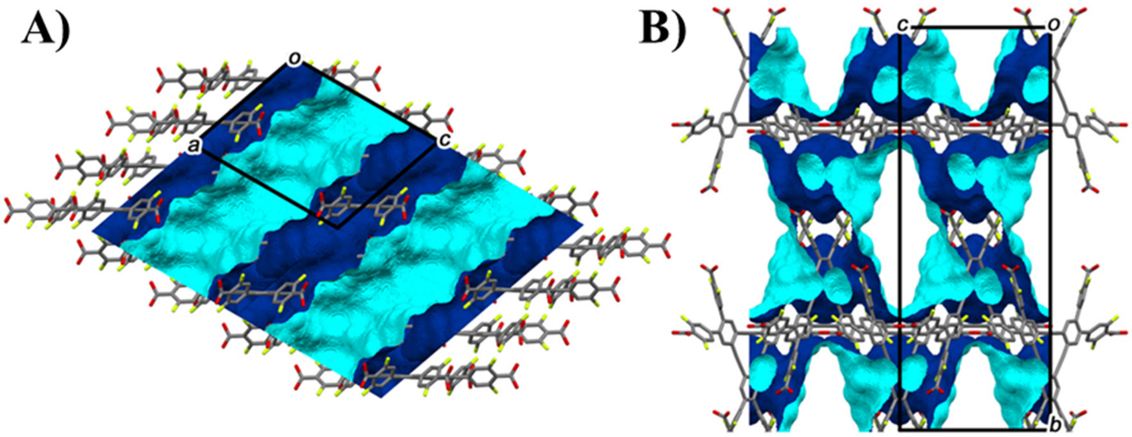

Figure S3. (**A**) and (**B**) Visualized contact surface of the channel viewed down from the *b* and *a* axes, respectively. The dimension of the aperture is 17 Å × 8 Å.

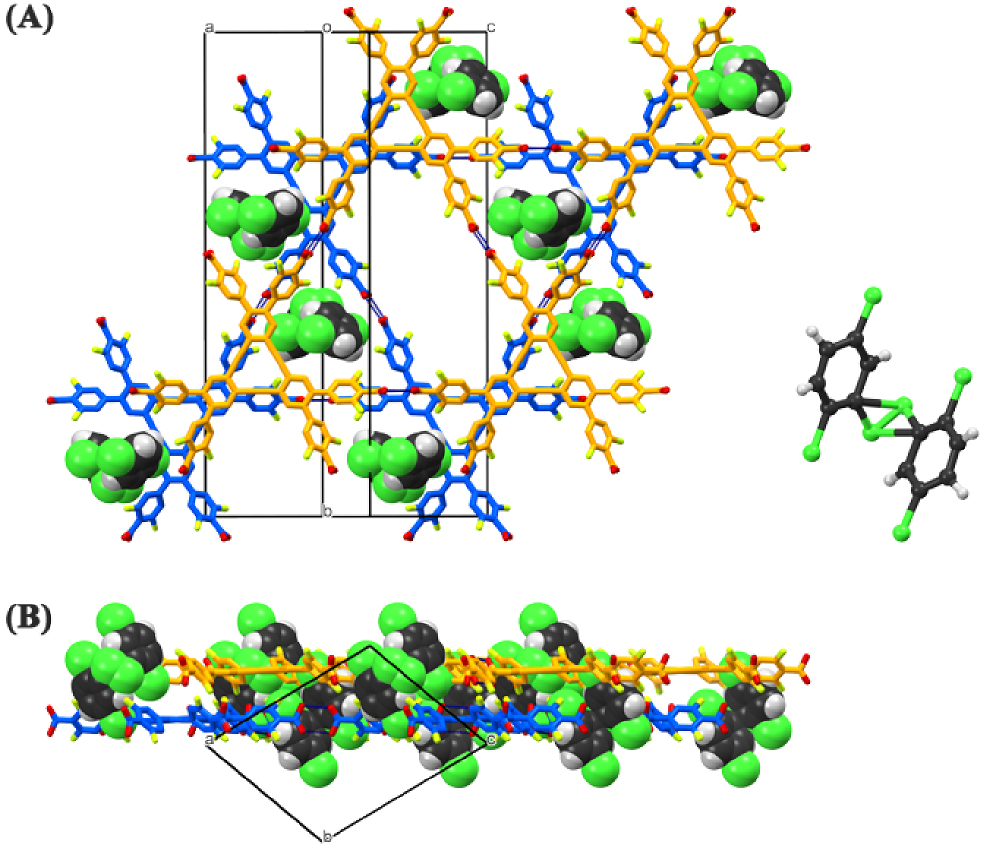

Figure S4. A selected framework of T12F-1(124TCB) with included solvent molecules. (**A**) A top and (**B**) side views of rhombic motifs. Inset: disordered molecule of 124TCB. The other solvent molecules are not solved due to severe disorder.

4. Optical Properties of the Compounds

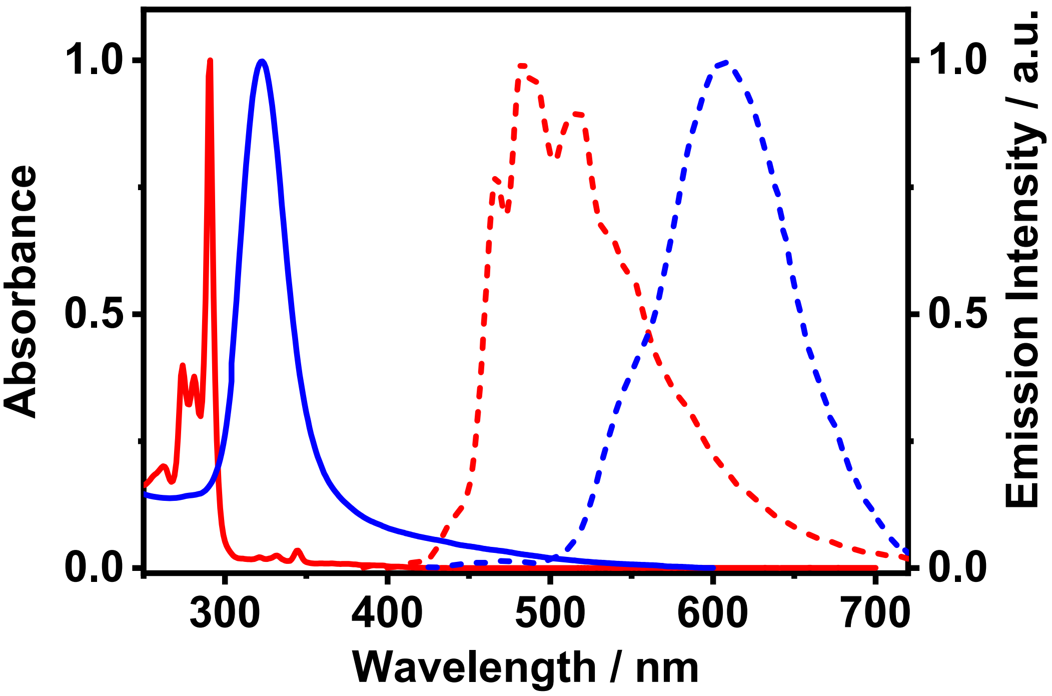

Figure S5. UV-visible absorption (solid lines) and emission spectra (dashed lines) of Nu-T12 (red) and T12-COOH (blue) in DMF solutions. The excitation wavelength for the emission spectra is 370 nm.

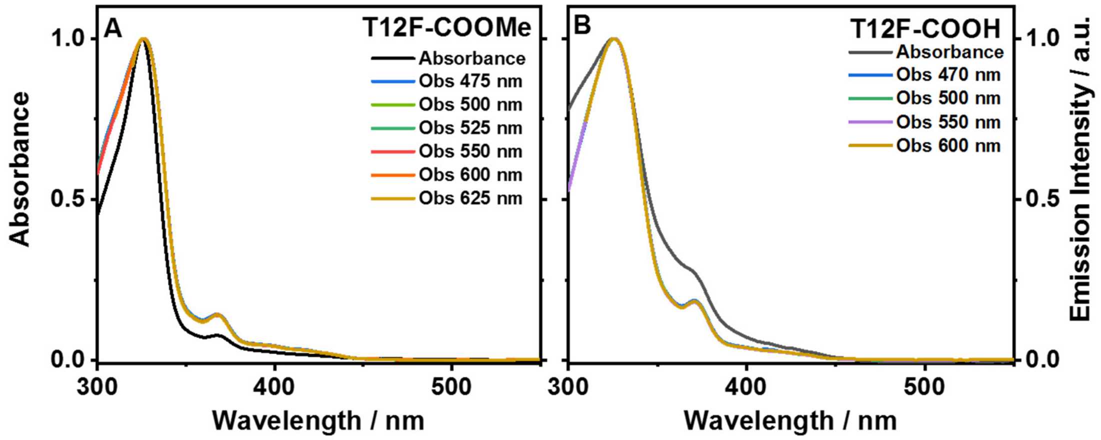

Figure S6. Normalized UV-visible absorption and excitation spectra of (**A**) T12F-COOMe and (**B**) T12F-COOH in DMF solutions. The observation wavelengths for the excitation spectra are indicated.

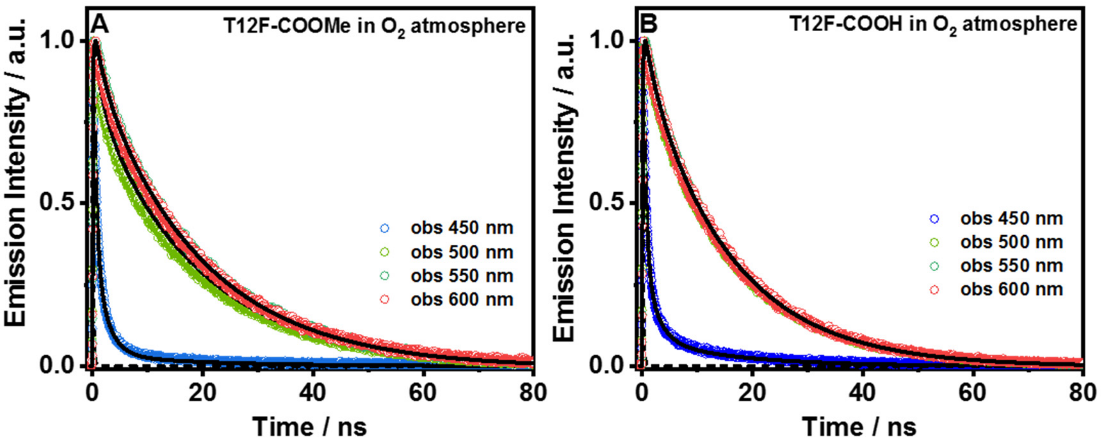

Figure S7. Normalized magic-angle emission decays of (**A**) T12F-COOMe and (**B**) T12F-COOH in DMF solutions under oxygen atmosphere upon excitation at 390 nm. The solid lines are from the best global fit using a multiexponential function. The dashed line is the IRF of the setup.

Table S2. Values of time constants (τ_i), normalized (to 100) pre-exponential factors (a_i) and contributions ($c_i = \tau_i \times a_i / \sum(\tau_i \times a_i)$) in the signal obtained from a global multiexponential fit of T12F-COOMe and T12F-COOH in DMF solutions under oxygen atmosphere. The excitation wavelength was 390 nm, and the observation wavelengths are indicated.

Sample	λ_{obs} (nm)	τ_1 (ps) ± 50 ps	A_1	c_1	τ_2 (ns) ± 0.2 ns	A_2	c_2	τ_3 (ns) ± 1.7 ns	A_3	c_3
T12F-COOMe	450	410	69	24	2.4	27	40	19.1	4	36
	500		17	1		13	3		70	96
	550		-	-		12	2		88	98
	600		-	-		12	2		88	98
T12F-COOH	450	400	69	17	2.0	24	26	15.6	7	57
	500		-	-		10	1		90	99
	550		-	-		10	1		90	99
	600		-	-		10	1		90	99

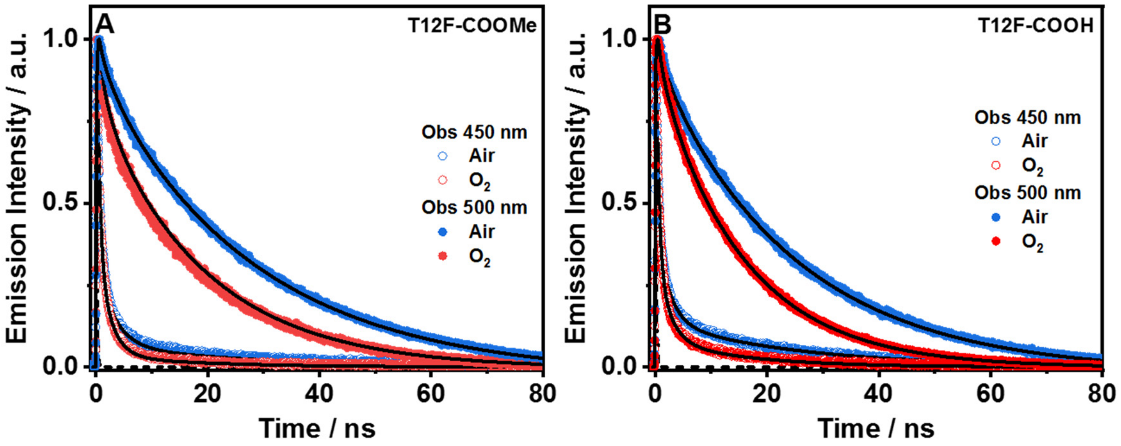

Figure S8. Representative magic-angle emission decays of (**A**) T12F-COOMe and (**B**) T12F-COOH in DMF solutions under ambient (blue decays) and oxygen (red decays) atmospheres upon excitation at 390 nm. The solid lines are from the best global fit using a multiexponential function. The dashed line is the IRF of the setup.

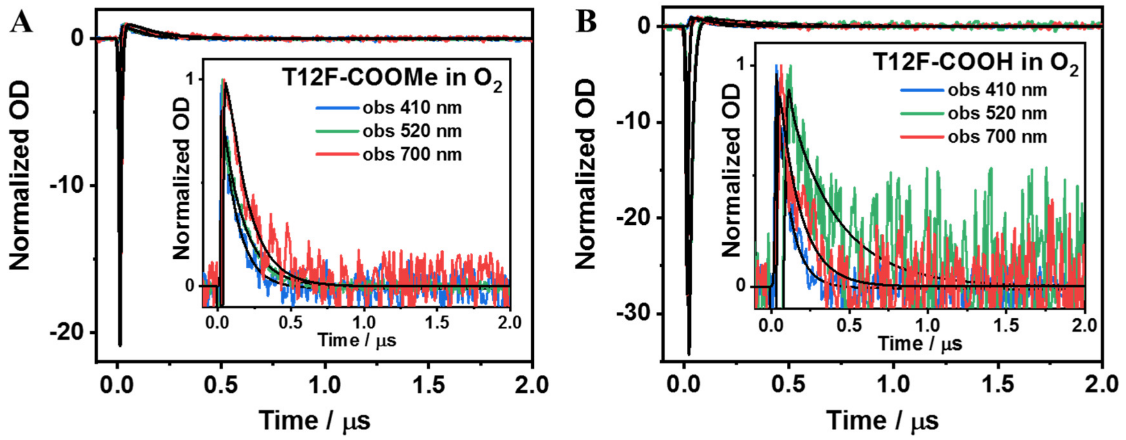

Figure S9. Transient decays of (**A**) T12F-COOMe and (**B**) T12F-COOH in DMF solution and under oxygen atmosphere upon excitation at 355 nm. The insets show a zoom of the positive transient decay. The solid lines are from the best global fit using a multiexponential function.

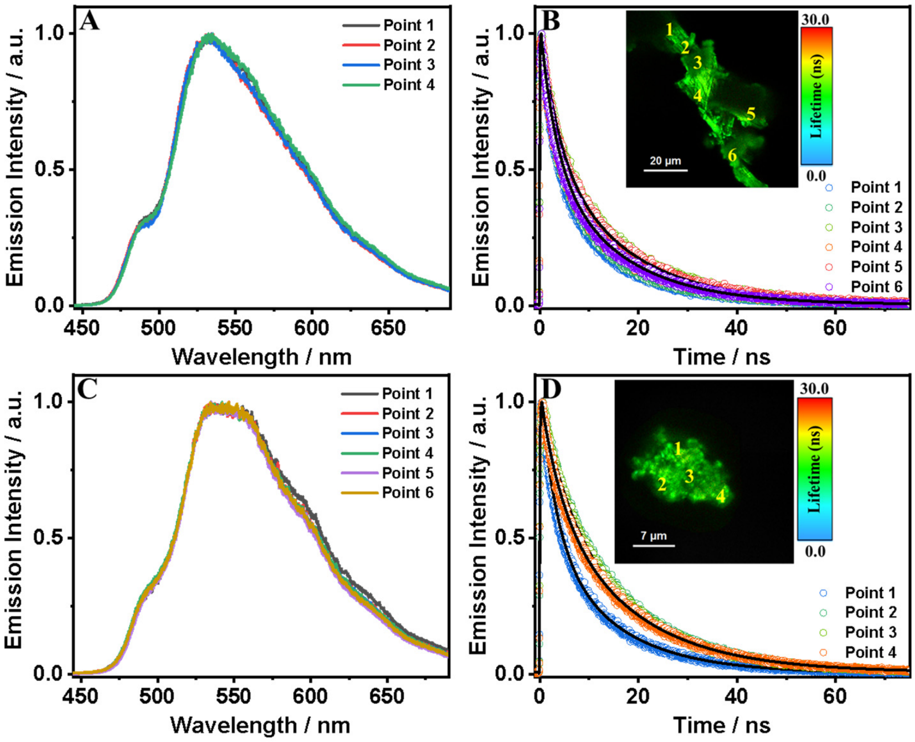

Figure S10. (**A**) and (**C**) Emission spectra, and (**B**) and (**D**) emission decays at selected points (indicated in the FLIM images) of T12F-ester. The excitation wavelength was 390 nm.

Table S3. Values of time constants (τ_i) and normalized (to 100) pre-exponential factors (a_i) obtained from the fit of the emission decays of the different points of T12F-ester. The fluorescence lifetime images of the related material are shown in Figure S17. The errors in the time constant values are about 15%.

A)

Point	τ_1 (ns)	a_1	τ_2 (ns)	a_2
**1**	2.8	49	13.0	51
**2**	2.8	47	13.2	53
**3**	3.1	40	13.8	60
**4**	2.9	45	13.5	55
**5**	3.2	41	13.8	59
**6**	3.0	45	13.5	55
**Average**	2.9	44	13.4	56

B)

Point	τ_1 (ns)	a_1	τ_2 (ns)	a_2
**1**	3.1	50	13.5	50
**2**	3.3	20	14.1	80
**3**	3.3	23	14.1	77
**4**	3.4	34	14.0	66
**Average**	3.2	32	13.9	68

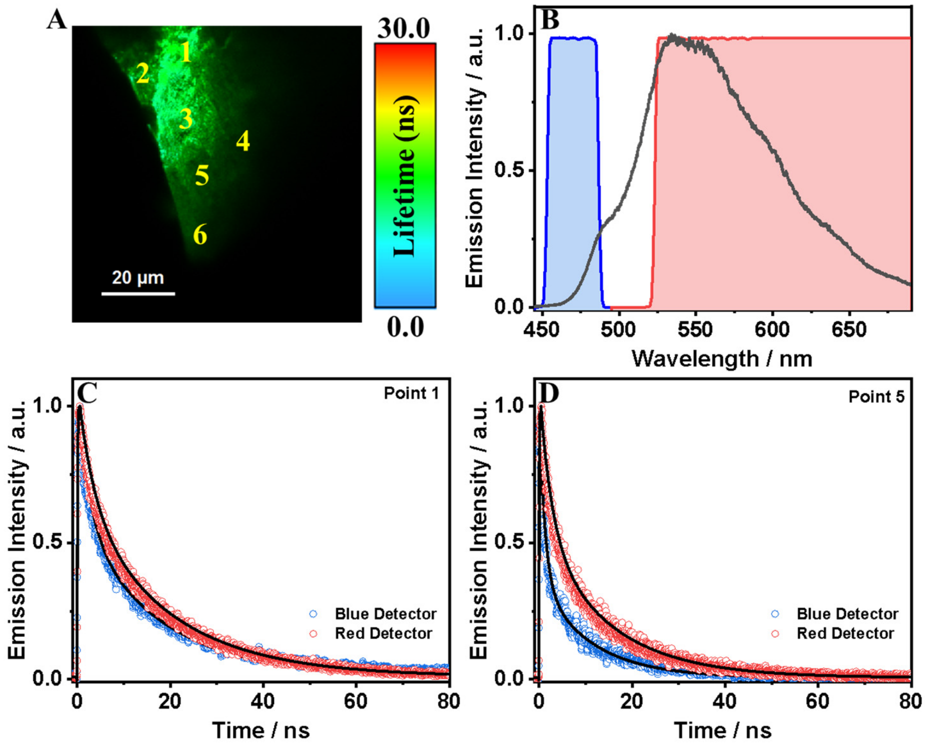

Figure S11. (**A**) FLIM image and (**B**) emission spectrum of T12F-ester. (**C**) and (**D**) Emission decays at selected spectral ranges using two different filters of transmission to gate at the blue and red regions as shown in (**B**). The solid lines are from the best-fit using a multiexponential function. The excitation wavelength was 390 nm.

Table S4. Values of time constants (τ_i) and normalized (to 100) pre-exponential factors (a_i) obtained from the fit of the emission decays of the different points and using different filters of T12F-ester shown in Figure S18. The errors in the time constant values are about 15%.

Point	Detector	τ_1 (ns)	a_1	τ_2 (ns)	a_2
1	Blue	2.7	44	16.0	56
	Red	3.0	38	16.0	62
2	Blue	2.0	50	13.0	50
	Red	3.1	48	14.0	52
3	Blue	2.5	48	15.0	52
	Red	3.0	48	15.0	52
4	Blue	2.0	55	13.0	45
	Red	3.1	49	16.0	51
5	Blue	2.0	60	13.0	40
	Red	2.6	49	15.0	51
6	Blue	2.3	60	15.0	40
	Red	2.5	46	15.0	54
Average	Blue	2.2	53	14.2	47
	Red	2.9	46	15.1	54

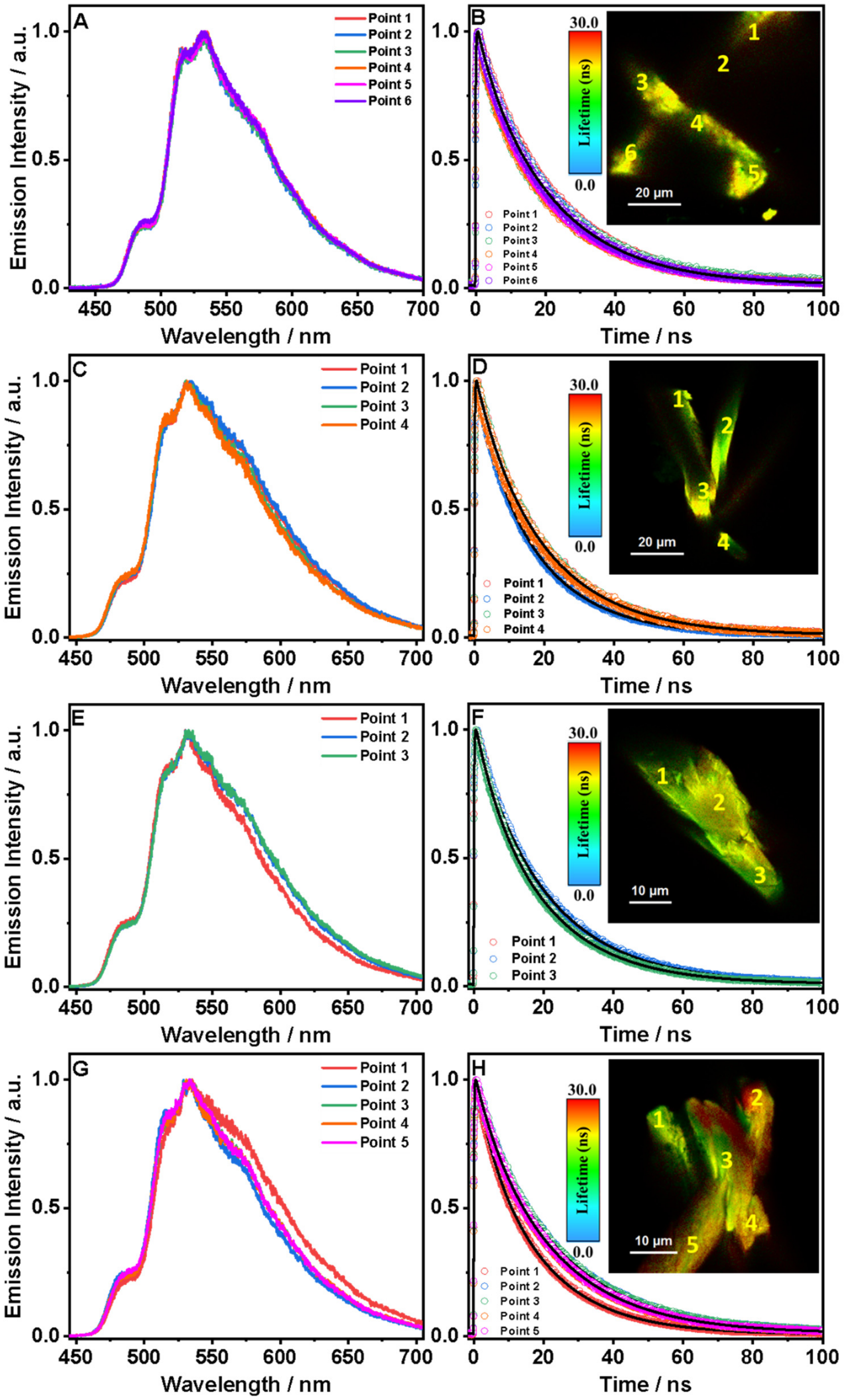

Figure S12. (**A**), (**C**), (**E**), (**G**) Emission spectra, and (**B**), (**D**), (**F**), (**H**) emission decays at selected points (indicated in the FLIM images) of T12F-1(124TCB) crystals. The excitation wavelength was 390 nm.

Table S5. Values of time constants (τ_i) and normalized (to 100) pre-exponential factors (a_i) obtained from the fit of the emission decays of the different points of T12F-1(124TCB) crystals. The fluorescence lifetime images of the related crystals are shown in Figure S19. The errors in the time constant values are about 15%.

Crystal in Figure	Point	τ_1 (ns)	a_1	τ_2 (ns)	a_2
B	1	3.9	3	22.1	97
	2	3.9	5	21.7	95
	3	4.0	11	21.5	89
	4	4.5	12	21.5	88
	5	4.6	7	21.3	93
	6	4.5	9	21.7	91
	Average	4.3	8	21.7	92
D	1	6.0	10	20.0	90
	2	5.8	16	19.0	84
	3	5.8	12	19.9	88
	4	6.0	11	20.0	89
	Average	5.9	12	19.8	88
F	1	5.8	9	20.0	91
	2	5.5	8	20.1	92
	3	5.6	12	19.6	88
	Average	5.6	10	19.9	90
H	1	5.1	15	19.0	85
	2	5.4	9	20.1	91
	3	5.4	8	20.2	92
	4	5.9	9	20.0	91
	5	5.9	10	20.0	90
	Average	5.6	10	19.8	90

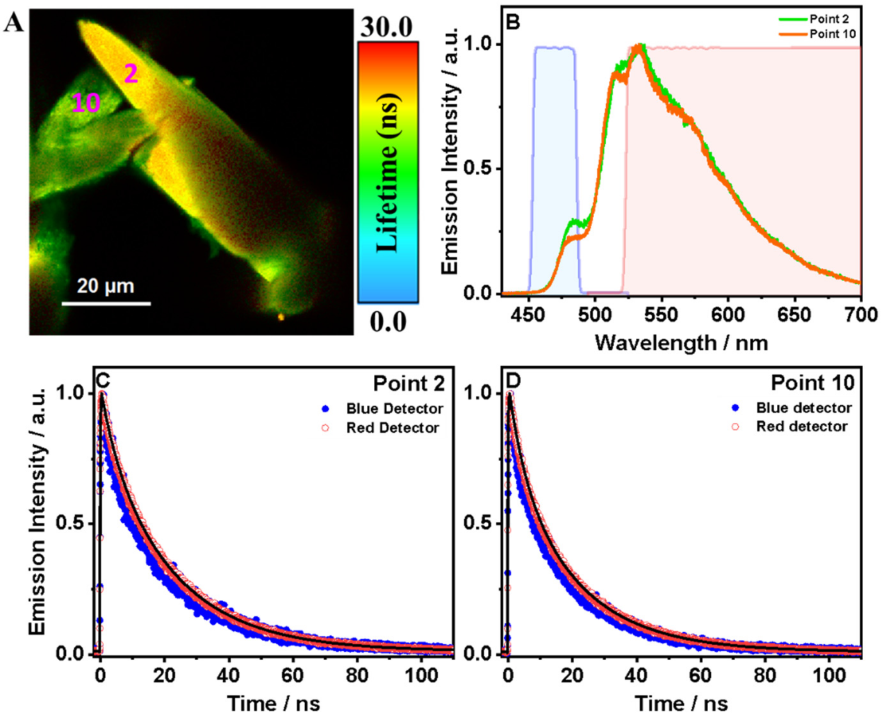

Figure S13. (**A**) FLIM image and (**B**) emission spectra of a T12F-1(124TCB) crystal. (**C**,**D**) Emission decays at a selected spectral range using two different filters of transmission to gate at the blue and red regions as shown in (**B**). The solid lines are from the best-fit using a multiexponential function. The excitation wavelength was 390 nm. The values from the fits are shown in the following table.

Table S6. Values of time constants (τ_i) and normalized (to 100) pre-exponential factors (a_i) obtained from the fit of the emission decays of the different points and using different filters of T12F-1(124TCB). The fluorescence lifetime image of the related crystal is shown in Figure S20. The errors in the time constant values are about 15%.

Point	Detector	τ_1 (ns)	a_1	τ_2 (ns)	a_2
2	Blue	4.4	22	21.4	78
	Red	4.9	16	22.0	84
10	Blue	4.5	15	20.0	85
	Red	5.0	13	20.8	87

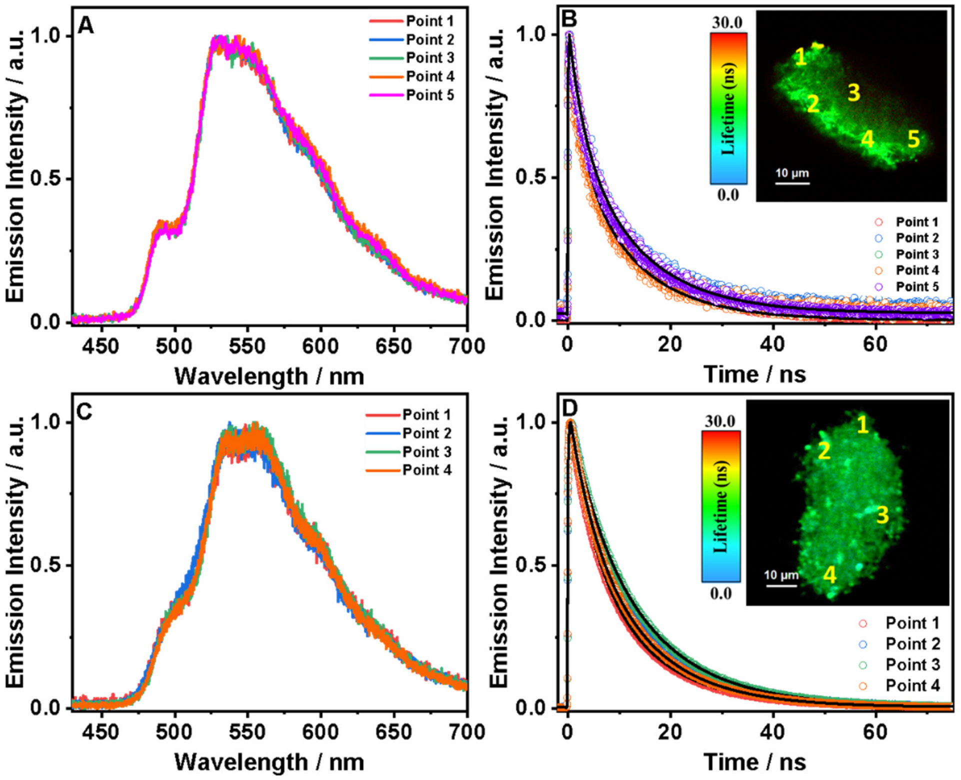

Figure S14. (**A**,**C**) Emission spectra and (**B**,**D**) emission decays at selected points (indicated in the FLIM images) of T12F-1(124TCB). The excitation wavelength was 390 nm.

Table S7. Values of time constants (τ_i) and normalized (to 100) pre-exponential factors (a_i) obtained from the fit of the emission decays of the different points of T12F-1(124TCB). The fluorescence lifetime images of the related material are shown in Figure S21. The errors in the time constant values are about 15%.

Crystal in Figure	Point	τ_1 (ns)	a_1	τ_2 (ns)	a_2
B	1	2.9	32	11.8	68
	2	3.0	37	12.0	63
	3	3.0	40	11.8	60
	4	2.9	40	12.0	60
	5	2.9	33	12.1	67
	Average	2.9	36	11.9	64
D	1	3.5	30	12.7	70
	2	3.9	22	13.1	78
	3	3.5	19	13.2	81
	4	3.5	28	12.6	72
	Average	3.6	25	12.9	75

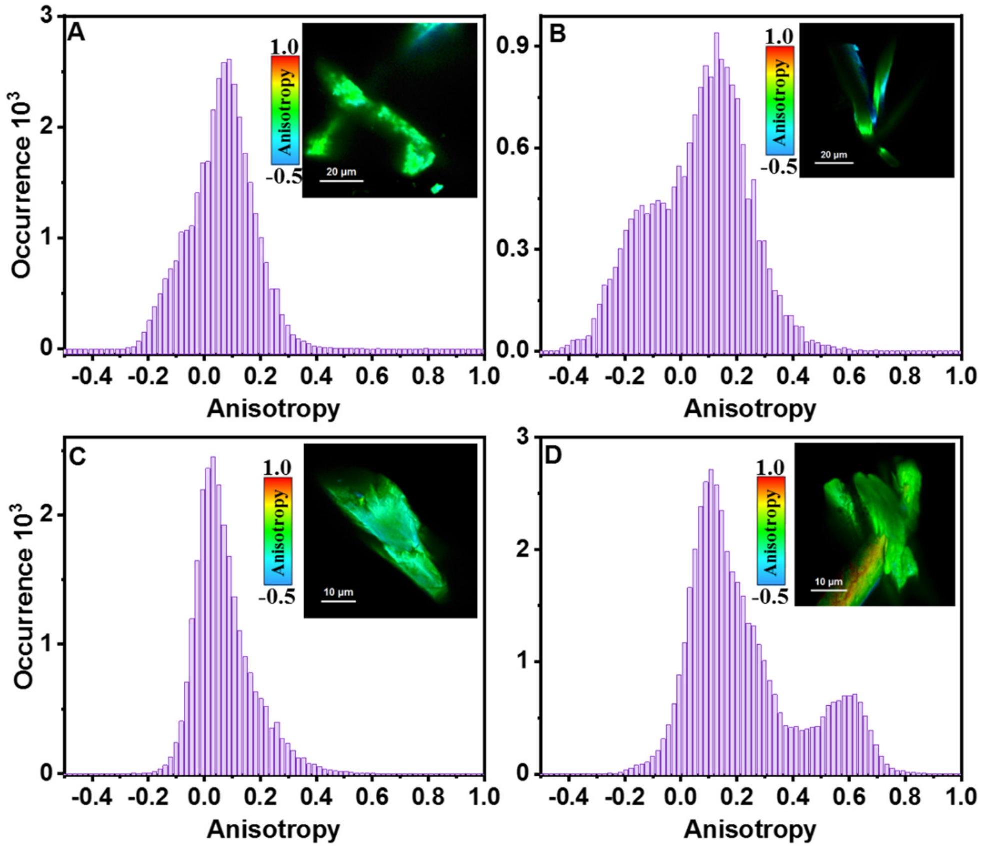

Figure S15. Histograms of the emission anisotropy of the related crystals of T12F-1(124TCB). The insets show the anisotropic images. The excitation wavelength was 390 nm.

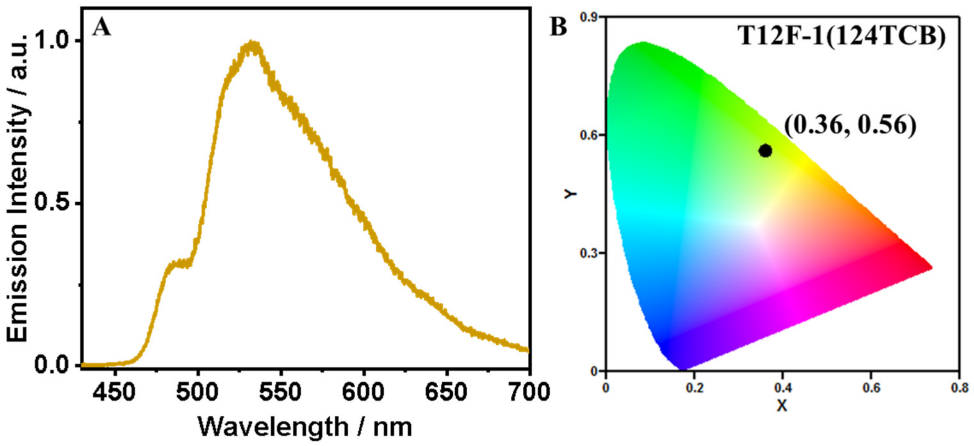

Figure S16. (**A**) Representative emission spectra and (**B**) Commission International De I'Eclairage coordinates of T12F-1(124TCB) crystals.

5. NMR Spectra of Synthesized Compounds

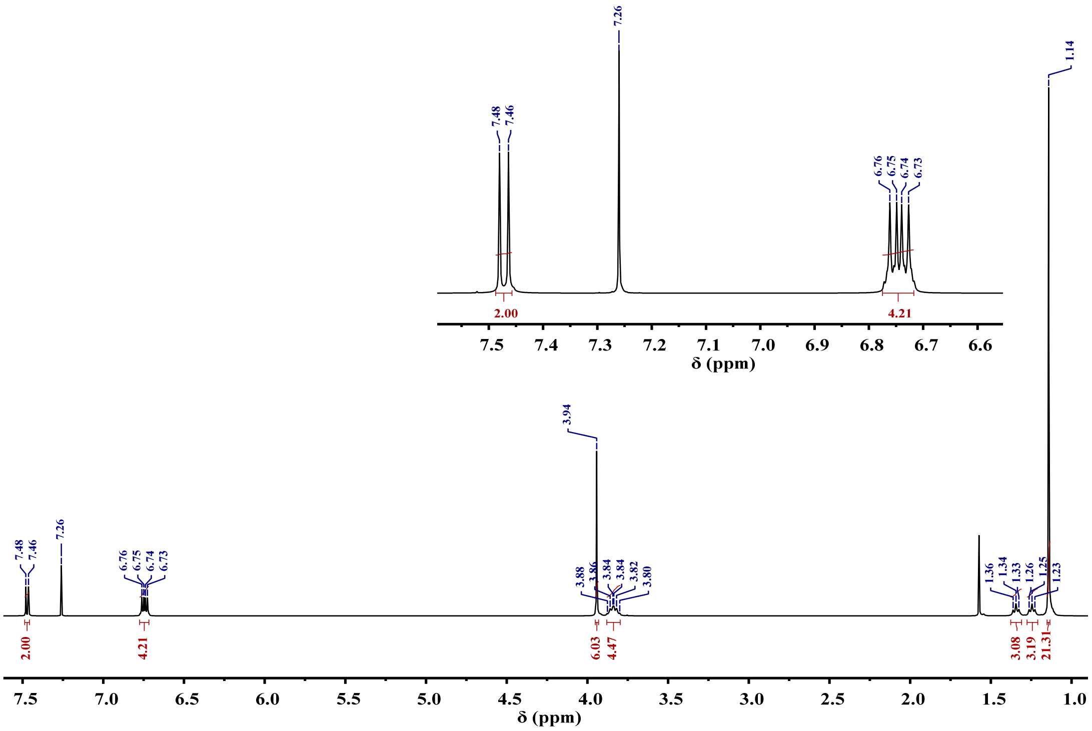

Figure S17. ^{1}H NMR (400 MHz, $CDCl_3$) spectrum of o-Terphenyl derivative **2.**

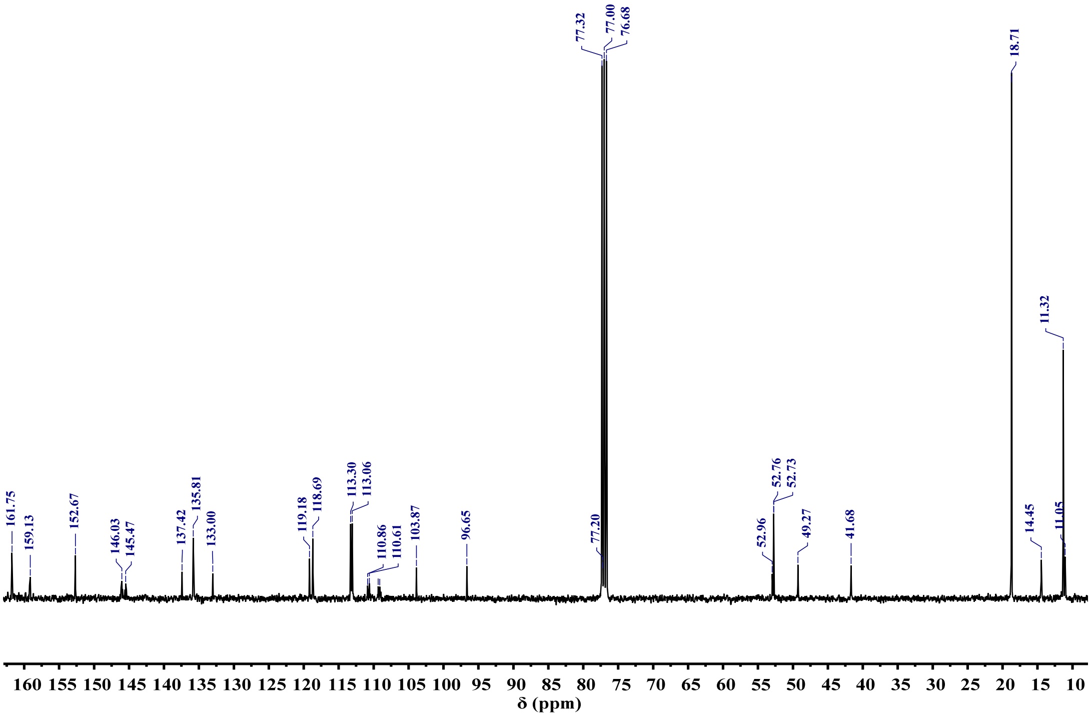

Figure S18. ^{13}C NMR (100 MHz, $CDCl_3$) spectrum of o-Terphenyl derivative **2.**

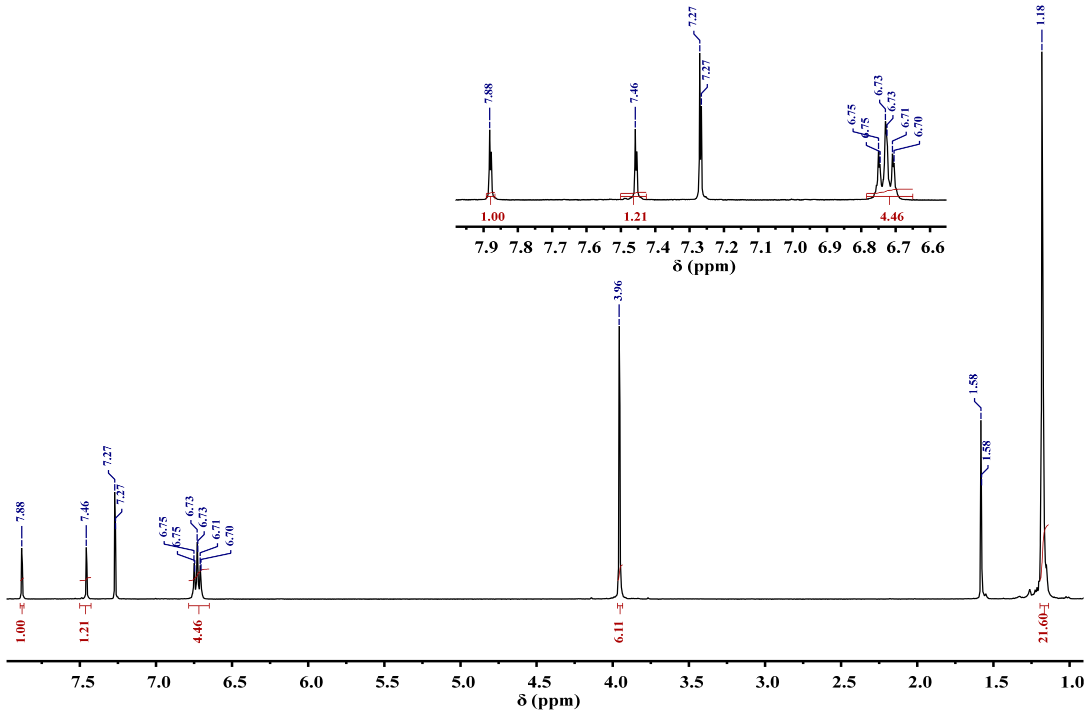

Figure S19. ^{1}H NMR (400 MHz, $CDCl_3$) spectrum of iodobenzene derivative **3.**

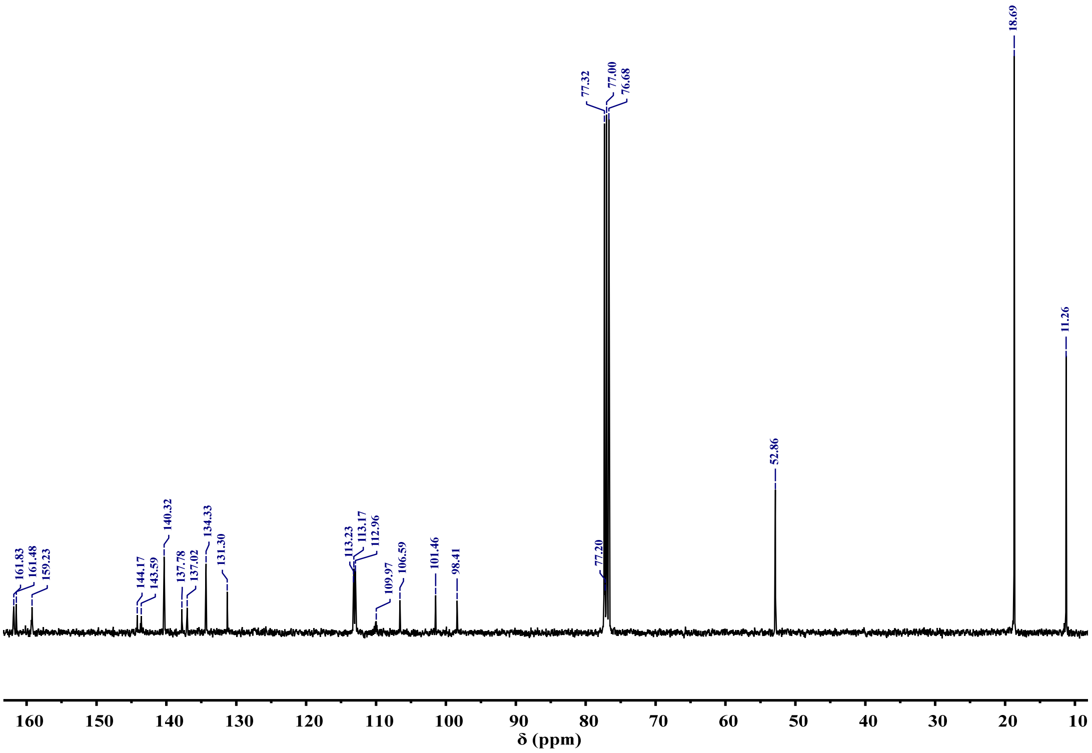

Figure S20. ^{13}C NMR (100 MHz, $CDCl_3$) spectrum of iodobenzene derivative **3.**

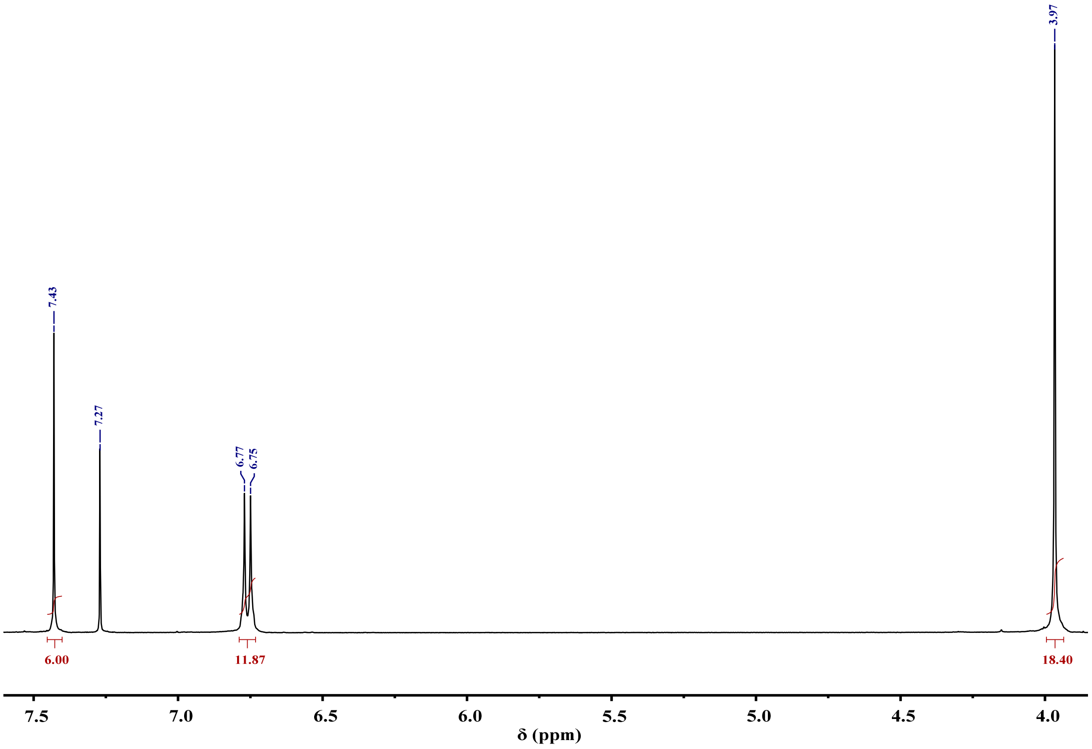

Figure S21. ^1H NMR (400 MHz, $CDCl_3$) spectrum of T12F-COOMe.

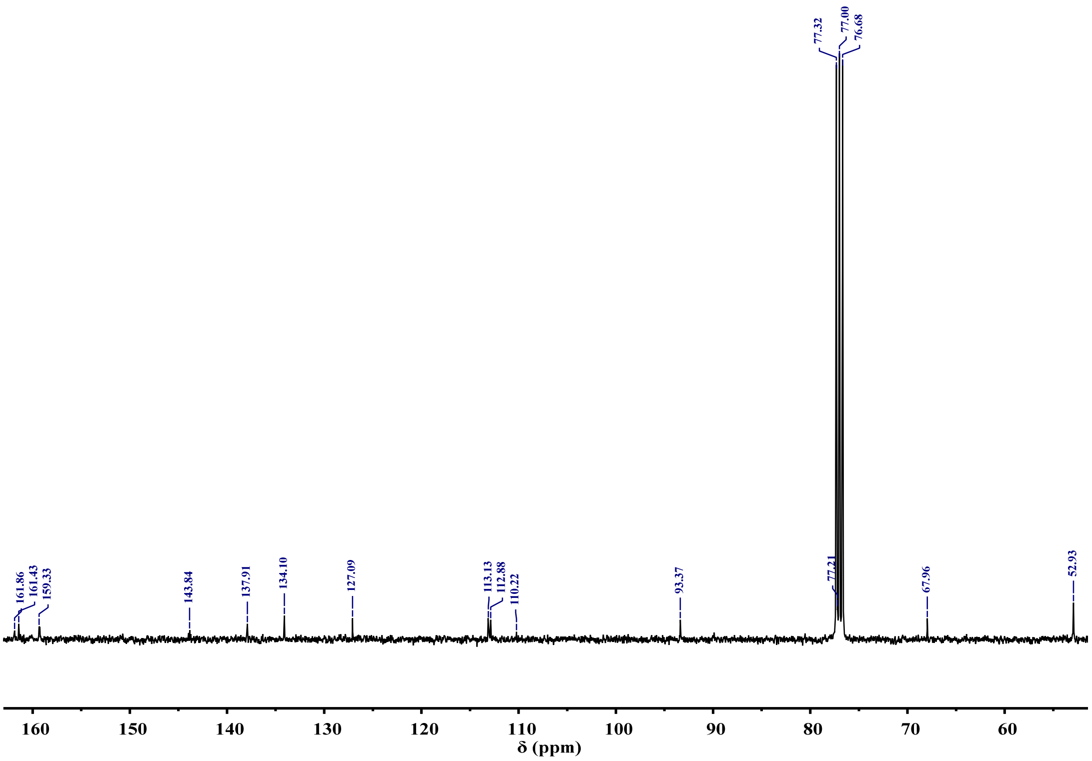

Figure S22. ^1H NMR (100 MHz, $CDCl_3$) spectrum of T12F-COOMe.

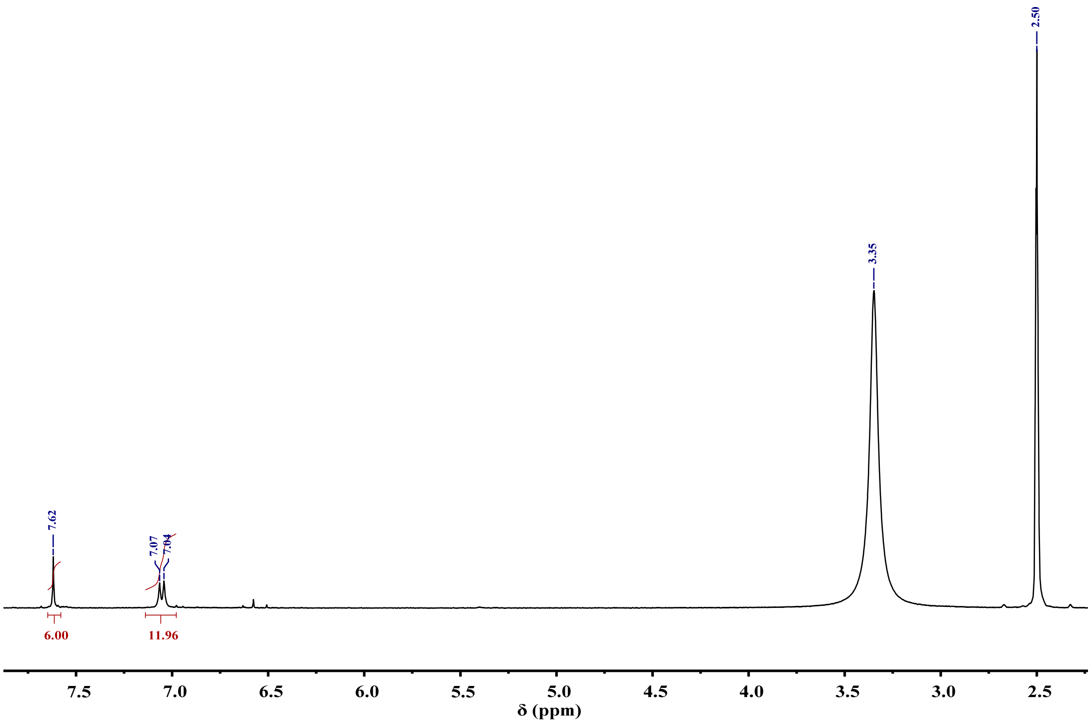

Figure S23. ^{1}H NMR (400 MHz, DMSO-d_6) spectrum of T12F-COOH.

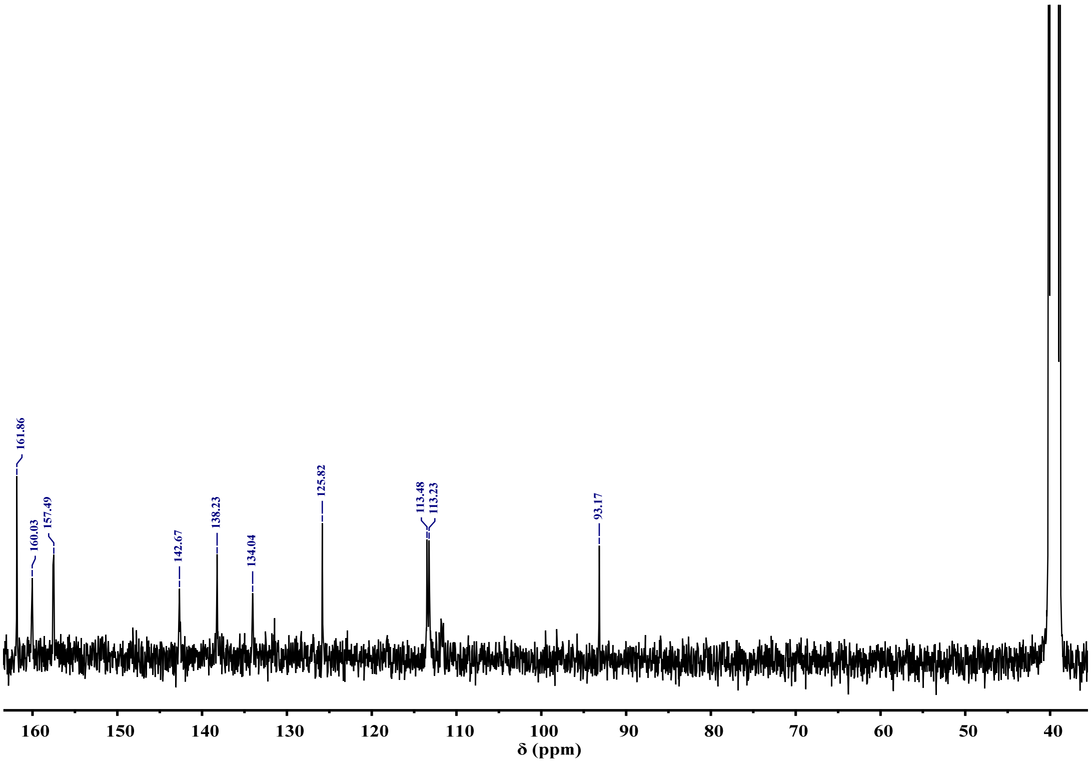

Figure S24. ^{13}C NMR (100 MHz, DMSO-d_6) spectrum of T12F-COOH.

References

1. Sheldrick, G. SHELXT - Integrated space-group and crystal-structure determination. *Acta Crystallogr. Sect. A* **2015**, *71*, 3-8, doi:doi:10.1107/S2053273314026370.
2. Sheldrick, G. Crystal structure refinement with SHELXL. *Acta Crystallogr. Sect. C* **2015**, *71*, 3-8, doi:doi:10.1107/S2053229614024218.
3. Van der Sluis, P.; Spek, A.L. BYPASS: an effective method for the refinement of crystal structures containing disordered solvent regions. *Acta Crystallogr. Sect. A* **1990**, *46*, 194-201, doi:doi:10.1107/S0108767389011189.
4. Spek, A. Structure validation in chemical crystallography. *Acta Crystallogr. Sect. D* **2009**, *65*, 148-155, doi:doi:10.1107/S090744490804362X.
5. Organero, J.A.; Tormo, L.; Douhal, A. Caging Ultrafast Proton Transfer and Twisting Motion of 1-Hydroxy-2-Acetonaphthone. *Chem. Phys. Lett.* **2002**, *363*, 409-414, doi:10.1016/s0009-2614(02)01233-2.
6. Randino, C.; Ziolek, M.; Gelabert, R.; Organero, J.A.; Gil, M.; Moreno, M.; Lluch, J.M.; Douhal, A. Photo-deactivation pathways of a double H-bonded photochromic Schiff base investigated by combined theoretical calculations and experimental time-resolved studies. *Phys. Chem. Chem. Phys.* **2011**, *13*, 14960-14972, doi:10.1039/C1CP21039D.
7. Schaffer, J.; Volkmer, A.; Eggeling, C.; Subramaniam, V.; Striker, G.; Seidel, C.A.M. Identification of Single Molecules in Aqueous Solution by Time-Resolved Fluorescence Anisotropy. *J. Phys. Chem. A* **1999**, *103*, 331-336, doi:10.1021/jp9833597.
8. Buschmann, V.; Bleckmann, A.; Bülter, A.; Krämer, B.; Nikolaus, J.; Schwarzer, R.; Korte, T.; Ziomkowska, J.; Herrmann, A.; Koberling, F. Polarization Extension Unit for LSM Upgrade Kits. *PicoQuant*.
9. Hisaki, I.; Nakagawa, S.; Ikenaka, N.; Imamura, Y.; Katouda, M.; Tashiro, M.; Tsuchida, H.; Ogoshi, T.; Sato, H.; Tohnai, N.; et al. A Series of Layered Assemblies of Hydrogen-Bonded, Hexagonal Networks of C3-Symmetric π-Conjugated Molecules: A Potential Motif of Porous Organic Materials. *J. Am. Chem. Soc.* **2016**, *138*, 6617-6628, doi:10.1021/jacs.6b02968.
